# Current Applications and Future Directions of Artificial Intelligence in Prostate Cancer Diagnosis: A Narrative Review

**DOI:** 10.3390/curroncol33030166

**Published:** 2026-03-13

**Authors:** Cong-Yi Zhu, Rui Qu, Yi Dai, Luo Yang

**Affiliations:** 1 West China School of Public Health and West China Fourth Hospital, Sichuan University, Chengdu 610041, China; 2021151650114@stu.scu.edu.cn; 2 Department of Urology and Pelvic Surgery and Andrology, West China School of Public Health and West China Fourth Hospital, Sichuan University, Chengdu 610041, China; daiyimed@163.com (Y.D.); yangluomed@163.com (L.Y.)

**Keywords:** prostate cancer, artificial intelligence, machine learning, deep learning, medical imaging, digital pathology, liquid biopsy, radiomics, biomarkers, computer-aided diagnosis

## Abstract

Prostate cancer (PCa) remains a major global health challenge, yet conventional diagnostic methods are often limited by suboptimal accuracy and efficiency. Artificial intelligence (AI) has emerged as a rapidly developing technology capable of integrating multi-source data to enhance clinical decision-making. This narrative review synthesizes current evidence regarding AI applications across key diagnostic domains, including medical imaging, digital pathology, liquid biopsy, and multi-omics integration. Findings indicate that AI models for magnetic resonance imaging (MRI) can improve risk stratification and may reduce unnecessary biopsies in some cohorts, particularly when evaluated alongside structured radiology assessment and clinical variables. In digital pathology, deep learning algorithms have shown high agreement with expert genitourinary pathologists for automated Gleason grading in controlled and externally validated settings, with potential to reduce reporting time for high-volume workflows. Additionally, AI-powered liquid biopsy models may support non-invasive risk stratification, particularly for patients with prostate-specific antigen (PSA) levels in the diagnostic gray zone, while multi-omics integration is being investigated to enhance personalized assessment. Despite advances, challenges regarding data heterogeneity, algorithm interpretability, and workflow integration persist. Future research should prioritize multimodal data fusion, explainable AI development, robust calibration and decision-analytic evaluation, and large-scale prospective validation to standardize protocols and fully realize the potential of AI in precision prostate cancer care.

## Simple Summary

Artificial intelligence is rapidly reshaping how prostate cancer is detected and characterized. Current diagnostic tools, including prostate-specific antigen testing, digital rectal examination, and magnetic resonance imaging, can lead to missed clinically significant cancers, unnecessary biopsies, and inconsistent interpretations across clinicians and institutions. This review summarizes recent applications of artificial intelligence in five diagnostic domains: medical imaging, digital pathology, liquid biopsy, multi-omics integration, and analysis of clinical information. Across selected tasks and clinical settings, artificial intelligence methods have been reported to improve diagnostic consistency, automate time-consuming tasks such as lesion detection and tumor grading, and support non-invasive risk stratification, particularly for men with borderline test results where biopsy decisions are difficult. The review also outlines key barriers to real-world adoption, including data heterogeneity, limited interpretability, workflow integration challenges, and regulatory and ethical concerns. Future efforts should prioritize multimodal data fusion with prespecified clinical endpoints, explainable models, and large prospective multicenter validation to enable safe, standardized clinical implementation.

## 1. Introduction

Prostate cancer (PCa) represents a significant global health burden, with 1.467 million new cases and 397,000 deaths reported worldwide in 2022, ranking as the fourth most common cancer and fifth leading cause of cancer-related mortality among men [1]. While early detection is crucial for improving patient outcomes [2], the current diagnostic pathway is fraught with limitations. Prostate-specific antigen (PSA) testing, the cornerstone of screening, suffers from poor specificity, leading to both overdiagnosis of indolent disease and missed diagnoses of aggressive cancers [3]. Digital rectal examination (DRE) is highly subjective and offers limited predictive value, particularly in men with low PSA levels [4]. Although magnetic resonance imaging (MRI) has significantly advanced PCa diagnosis, it is still hampered by false-positive and false-negative results, inter-reader variability, and high costs [5]. Furthermore, emerging biomarkers such as circulating tumor DNA (ctDNA) and microRNAs (miRNAs) face challenges related to low abundance, insufficient specificity, and a lack of standardized protocols, limiting their widespread clinical adoption [6]. These collective challenges underscore an urgent need for more accurate, efficient, and non-invasive diagnostic strategies.

Beyond accuracy metrics, the clinical problem in PCa diagnosis is fundamentally one of decision calibration: identifying men who truly need biopsy or definitive treatment while safely sparing those with indolent disease. This requires not only detecting lesions, but also estimating clinically meaningful endpoints such as clinically significant PCa (csPCa) probability, tumor grade group, lesion volume, extracapsular extension risk, and uncertainty in borderline scenarios. In this context, the “diagnostic pathway” is a sequence of interdependent steps (screening, imaging acquisition, image interpretation, targeted/systematic biopsy, histologic grading, and longitudinal monitoring), where errors and variability compound across steps. AI is increasingly studied as a method to reduce this cumulative variability, quantify uncertainty, and provide risk estimates that can be incorporated into shared decision-making.

Artificial intelligence (AI), particularly machine learning (ML) and deep learning (DL), offers a framework to transform heterogeneous diagnostic signals into probabilistic and reproducible clinical outputs [7]. Traditional ML typically relies on expert-defined features (e.g., radiomics, handcrafted morphometrics, or curated biomarker panels) and is advantageous when sample sizes are limited or when interpretability and feature attribution are prioritized. DL models can learn hierarchical representations directly from raw inputs (MRI volumes, Positron emission tomography/computed tomography (PET/CT), whole-slide imaging (WSI) tiles, spectroscopy, or longitudinal laboratory trajectories), enabling end-to-end detection, segmentation, grading, and outcome prediction [8]. However, reproducibility and clinical trust depend on transparent reference standards, patient-level data partitioning, external validation, and reporting beyond discrimination (e.g., calibration and decision-curve analysis).

This review aims to critically evaluate the current applications of AI in PCa diagnosis, discuss its clinical value and limitations, and project its future trajectory, providing a comprehensive overview for clinicians and researchers.

## 2. Methods

### 2.1. Study Design

This manuscript is a narrative review aimed at synthesizing clinically relevant evidence on artificial intelligence applications for prostate cancer diagnosis across multiple diagnostic domains (medical imaging, digital pathology, liquid biopsy, multi-omics integration, and clinical information). We adopted a pre-specified, non-systematic method rather than a formal systematic review.

### 2.2. Data Sources and Search Strategy

We conducted the search for literature addressing the application of AI in prostate cancer diagnosis. Relevant studies published between January 2020 and September 2025 were identified through searches of PubMed and Web of Science. The search strategy incorporated key terms related to AI technologies and diagnostic modalities, including “prostate cancer,” “artificial intelligence,” “machine learning,” “deep learning,” “medical imaging,” “digital pathology,” “liquid biopsy,” “radiomics,” “biomarkers,” and “computer-aided diagnosis.” These terms were used in various combinations to capture a broad yet clinically relevant spectrum of publications. To facilitate accurate interpretation and critical appraisal, the review was limited to articles and reviews published in English. Given the rapidly evolving nature of AI-related technologies, particular emphasis was placed on original studies published within the most recent 5 years.

### 2.3. Eligibility Criteria (Inclusion and Exclusion)

**Figure 1:**
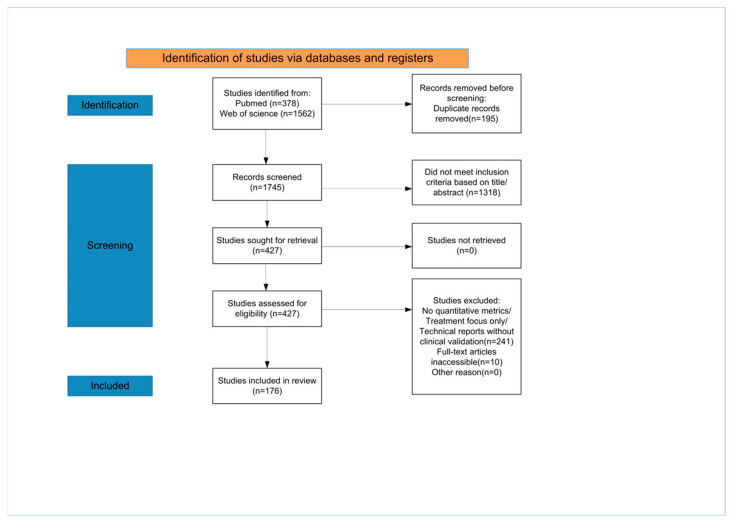
PRISMA flow diagram of the literature search and selection process. The diagram illustrates the number of records identified from PubMed and Web of Science, screened, and finally included.

Study selection was guided by clinical relevance, methodological quality, and potential implications for diagnostic decision-making, risk stratification, and patient outcomes in prostate cancer care. Given the narrative design, the PRISMA-style flow diagram (Figure 1) is presented as a transparent description of the screening process rather than as evidence of a fully systematic review. In the main text, references were prioritized for original research articles involving diagnostic models and studies with larger sample sizes. The complete list of all 176 included studies is available in Supplementary Table S1.

### 2.4. Inclusion Criteria

Eligible studies comprised original research articles, including randomized controlled trials, prospective validation studies, and retrospective cohort analyses, as well as systematic reviews published within the past five years. Studies were required to investigate the application of artificial intelligence approaches, including machine learning, deep learning, and computer-aided diagnosis (CAD), in prostate cancer diagnosis, Gleason grading, detection of clinically significant prostate cancer, or risk stratification. Acceptable technical domains included medical imaging modalities (MRI, PET/CT, and ultrasound), digital pathology, and liquid biopsy or multi-omics platforms. Only full-text articles published in English were considered.

### 2.5. Selection Priorities

To enhance clinical interpretability, preference was given to studies reporting quantitative diagnostic performance metrics, such as area under the receiver operating characteristic curve, sensitivity, specificity, accuracy, and F1-score. Particular attention was paid to investigations incorporating independent external validation cohorts to reduce the risk of overfitting and improve generalizability. Studies benchmarking AI performance against human experts or established clinical guidelines were considered methodologically robust and were therefore emphasized in the synthesis.

### 2.6. Exclusions

Studies were excluded if they focused exclusively on therapeutic interventions, surgical navigation, or treatment planning without reporting diagnostic performance outcomes. Technical reports and pilot studies lacking clinical validation or a clearly defined reference standard were excluded. Publications that did not provide quantitative diagnostic metrics were also omitted. Editorials, letters, conference abstracts without accessible full texts, case reports, duplicate datasets, and non-English publications were excluded to maintain methodological consistency.

### 2.7. Data Extraction and Synthesis Framework

Data extraction was conducted to enable a structured qualitative synthesis of the available evidence. Representative studies were selected for detailed evaluation with key characteristics summarized in relevant sections.

We prioritized studies with clinically aligned endpoints (e.g., csPCa detection, biopsy decision support, lesion-level localization linked to histopathology, Gleason/International Society of Urological Pathology (ISUP) grading support with patient-level validation, or biomarker-based diagnostic stratification).

Methodological quality signals were operationalized as: clear reference standard definition (e.g., histopathology from biopsy or prostatectomy where applicable), transparent dataset reporting (size and source), validation design descriptions (internal vs. external; single-center vs. multicenter; retrospective vs. prospective), and reporting of performance metrics (e.g., Area Under the Curve (AUC), sensitivity, specificity) and uncertainty (e.g., 95% confidence intervals (CI)) when available.

For each included study, we extracted and summarized: (1) diagnostic domain and task (detection, grading, risk stratification, subtype prediction); (2) data modality (MRI, PET/CT, ultrasound, WSI, blood/urine biomarkers, multi-omics, clinical data); (3) dataset characteristics (sample size, number of centers, public vs. institutional data); (4) reference standard; (5) validation strategy (internal split/cross-validation, external validation, prospective cohort, and/or reader study); (6) key performance results as reported by the authors. When studies differed in endpoint definitions (e.g., definitions of clinically significant PCa), we retained the study’s original definition and noted heterogeneity in the synthesis.

### 2.8. Consideration of Risk of Bias

Although a formal risk-of-bias tool (e.g., QUADAS-2) was not systematically applied across all included studies, potential sources of bias were explicitly considered throughout study selection and evidence synthesis.

Methodological strength was assessed in relation to external validation, given its importance in mitigating overfitting to single-center datasets and enhancing generalizability. The reliability of reference standards was critically examined, particularly with respect to whether radical prostatectomy histopathology or biopsy findings served as ground truth. Studies employing clearly defined and clinically robust reference standards were regarded as methodologically stronger.

Attention was also given to the handling of class imbalance between cancer and non-cancer cases, as disproportionate distributions may artificially inflate performance metrics. Furthermore, issues related to dataset heterogeneity and the limited interpretability of certain “black box” AI models were considered in the context of clinical implementation and reproducibility.

Rather than relying solely on checklist-based appraisal, emphasis was placed on external validity, transparency in methodological reporting, and clinical interpretability as guiding principles in evaluating the overall strength of evidence.

## 3. Artificial Intelligence in Medical Imaging: Capabilities and Constraints

Medical imaging is the bedrock of modern PCa diagnosis, and the integration of AI is fundamentally transforming how images are interpreted and utilized.

### 3.1. AI Applications in Magnetic Resonance Imaging (MRI): Performance and Persistent Challenges

MRI, especially multiparametric MRI (mpMRI), is the most mature and widely explored domain for AI in PCa diagnosis. Early research focused on combining radiomics with traditional ML algorithms. By extracting high-dimensional features—such as texture and shape, which are imperceptible to the human eye—and analyzing them with models like Logistic Regression (LogReg), Random Forest (RF), and Support Vector Machines (SVM) [9,10,11], numerous studies have demonstrated that these models can improve discrimination compared with the Prostate Imaging-Reporting and Data System (PI-RADS) score alone in predicting csPCa (Gleason Score ≥ 7, corresponding to ISUP Grade Group ≥ 2), with AUCvalues often reported in the 0.86–0.90 range [12,13]. Because PI-RADS is a categorical human assessment and radiomics/DL models often use different endpoints and operating points, comparisons should be interpreted in the context of cohort composition, prevalence, and reference standards.

The advent of DL, particularly Convolutional Neural Networks (CNNs) and their variants (e.g., 3D-CNNs, ResNet, U-Net), marked a paradigm shift from “feature engineering” to “end-to-end” learning [14,15,16,17]. DL models can learn diagnostic information directly from raw MRI scans, demonstrating competitive performance in tumor classification, segmentation, and automated detection across several validation cohorts [18,19,20,21,22,23,24]. For instance, the CorrSigNIA framework, which integrates pathological and imaging features, achieved an 80% accuracy in cancer subtyping [25]. In reader studies and multicenter evaluations, AI-powered computer-aided diagnosis (DL-CAD) systems may improve radiologist performance and/or reduce inter-reader variability, although the magnitude of benefit depends on case mix, reader experience, and prespecified thresholds [26]. In confirmatory paired designs (e.g., PI-CAI), AI systems have demonstrated performance that can be comparable to or noninferior to radiologists under specific evaluation protocols, supporting a potential role as an adjunct rather than a replacement [27,28,29]. The core data categories extracted from selected key studies are summarized in Table 1.

Comparative Analysis and Limitations:

Traditional ML and DL serve distinct yet complementary roles in MRI analysis. ML models offer better interpretability, allowing clinicians to understand which radiomic features contribute to a prediction. However, their performance is heavily dependent on the quality of manual feature extraction. DL models, with their powerful automated feature learning, often achieve higher accuracy but are frequently criticized for their “black box” nature, which can be a barrier to clinical trust [30]. For clinical deployment, interpretability should be considered alongside calibration, failure modes (e.g., small anterior lesions), and robustness to scanner/protocol variation.

The core value of AI in MRI extends beyond simply achieving a higher AUC, with its clinical potential manifesting primarily in two distinct areas. First, AI plays a crucial role in optimizing biopsy decisions. By accurately identifying csPCa, AI can improve the characterization of equivocal PI-RADS 3 lesions, thereby safely reducing the number of unnecessary biopsies and their associated complications and costs [31,32]. Furthermore, AI significantly enhances diagnostic efficiency and standardization. By automating time-consuming tasks such as lesion segmentation and measurement, AI reduces reading times and empowers less-experienced radiologists with expert-level assistance. Consequently, this promotes diagnostic homogenization across different healthcare institutions, particularly when integrated into Picture Archiving and Communication Systems (PACS) workflows and validated under local acquisition protocols [33,34].

However, significant limitations persist. First, model generalizability remains the greatest challenge. Models that perform exceptionally well on internal data often see a performance drop on external, multicenter datasets due to variations in MRI scanners, protocols, and patient populations [35,36,37,38]. Second, the sensitivity of AI models to small or atypically located lesions needs further improvement [39]. Finally, the seamless integration of AI tools into existing PACS and clinical workflows is a critical engineering hurdle that must be overcome for real-world implementation [40]. Post-deployment monitoring (drift detection, recalibration, and human override pathways) should be planned as part of implementation, particularly in multicenter health systems.

In addition, the AI potential in quantitative MRI analysis has grown substantially, particularly in leveraging parameter maps (such as apparent diffusion coefficient(ADC) values) and standardized quantitative features for cancer risk estimation. However, these quantitative approaches face significant challenges from scanner-to-scanner and protocol variability, requiring robust mitigation strategies including data normalization techniques, ComBat-style harmonization methods, multisite training protocols, and emerging domain adaptation approaches. Despite these technical developments, performance degradation across different imaging centers remains a persistent challenge, prompting investigation into solutions such as unsupervised domain adaptation to address dataset shift issues that continue to limit real-world implementation. Across all these applications, the translation of AI into clinical practice must carefully consider both the technical capabilities and the practical constraints that affect real-world performance.

**Table 1:** The core data categories extracted from selected key studies of prostate cancer diagnosis on MRI.

Study (Ref.)	Objective & AI Model	MRI Protocol & Analysis Level	Dataset & Reference Standard	Validation Strategy	Key Performance & Clinical Utility
Liu et al. (2024) [19]	Grading: Hybrid model (Radiomics + CNN with CPCB and SE-Net modules) for binary classification of prostate cancer Gleason score (GS ≤ 3 + 4 vs. GS ≥ 4 + 3).	mpMRI (T2-weighted imaging (T2WI), ADC), 3.0T, 3 mm slice thickness analysis: Lesion-level (3D tumor ROI with 5 mm perilesional margin)	*N* = 650 (Two hospitals: GPPH *n* = 408, FAHGMU *n* = 242) Ref: Histopathology (biopsy/surgical GS grading)	Multi-center retrospective study; Training set (*n* = 520), Testing set (*n* = 130)	Accuracy: 0.907; F1-score: 0.918; Recall: 0.944; Precision: 0.893; AUC: 0.946
Saha et al. (2024) [27]	Detection: Deep learning ensemble model (five top-performing algorithms) for detecting clinically significant prostate cancer (Gleason grade group ≥ 2).	bpMRI(Biparametric magnetic resonance imaging) Analysis: Case-level and lesion-level (0–100 likelihood score)	*N* = 10,207 MRI examinations (9129 patients); Training: 9207 cases; Testing: 1000 cases (400 for reader study) Ref: Histopathology (biopsy/prostatectomy) with ≥3 years follow-up (median 5 years)	External validation (Multi-center, international: Netherlands and Norway); Reader study with 62 radiologists from 45 centers in 20 countries	AUC: 0.91 (95% CI 0.87–0.94) vs. radiologists’ AUC 0.86 (0.83–0.89)—superior performance (*p* < 0.0001) At matched specificity (57.7%): 6.8% more csPCa detected, 50.4% fewer false positives, 20.0% fewer indolent cancers detected vs. mean radiologist performance
Sowmya et al. (2024) [21]	Classification: Taylor_AMFSOpt-DAttNNet (Deep Attention Neural Network with Adaptive Swarm Intelligence) for prostate cancer classification and severity estimation (high-grade vs. low-grade).	MRI (T2WI) Analysis: Image-level	*N* = 620 (Kaggle dataset: 440 training, 180 testing; external validation on SPIE-AAPM-NCI dataset with 346 patients) Ref: Histopathology (implied from dataset labels)	Internal validation with train–test split; External validation on SPIE-AAPM-NCI dataset	Accuracy: 97.8%; Precision: 97.9%; Recall: 96%; F1-score: 95%; Specificity: 94%
Zaridis et al. (2024) [23]	Segmentation: ProLesA-Net (multi-channel 3D architecture with MSSE and MSAG attention mechanisms) for prostate lesion segmentation.	bpMRI (T2W, ADC, Diffusion-weighted imaging (DWI) Analysis: Lesion-level (3D volumetric segmentation)	Training: PICAI dataset (*N* = 219 patients, 15,768 images); External testing: Prostate-158 dataset (*N* = 82 patients, 5904 images) Ref: Expert-annotated ground truth masks	External validation (multi-center: 3T Siemens and Philips scanners); compared against six DL models (3D U-Net, VNet, TransU-Net, USE-Net, nnU-Net, Attention U-Net)	Dice score: 30.56%; Recall: 33.91%; Precision: 33.72%; Hausdorff distance: 19.61 mm; Average surface distance: 4.43 mm For small lesions (<15 mm): Dice score: 22.33%; Recall: 27.54% For intermediate lesions (15–30 mm): Dice score: 39.63%; Hausdorff distance: 14.43 mm
Sanjid et al. (2024) [24]	Segmentation: HUNet (dual-pathway multi-scale hierarchical upsampling network) for prostate zonal segmentation (anatomical zones: transition zone, peripheral zone; tumor segmentation).	mpMRI (T2WI, DWI, ADC, Dynamic contrast-enhanced (DCE)) Analysis: Lesion-level and zonal-level (whole gland, anatomy, tumor)	ProstateX: *N* = 193 patients (163 training, 15 validation, 15 testing); Prostate158: *N* = 158 patients (128 training, 15 validation, 15 testing) Ref: Expert-annotated ground truth masks	Internal validation with train–test split; compared against U-Net and DenseNet baselines	ProstateX anatomy: Intersection over Union (IoU): 0.8449; Dice Similarity Coefficient (DSC): 0.9872; RAVD: 0.0944; ASSD: 0.0106 Prostate158 anatomy: IoU: 0.8065; DSC: 0.9831; RAVD: 0.0020; MHD: 3.5736 mm; ASD: 0.0070 mm Prostate158 tumor: DSC: 0.9974; MHD: 0.5205 mm
Cai et al. (2024) [31]	Detection: 3D CNN-based deep learning model to predict csPCa using patient-level labels without tumor location information; combined with clinical data (PSA, PSA density) and radiologist PI-RADS scores.	mpMRI (T2WI, DWI, ADC, DCE) Analysis: Patient-level (case-level) with Grad-CAM localization	Internal: *N* = 5735 examinations (5215 patients) from Mayo Clinic; External: ProstateX *N* = 204 Ref: Histopathology (biopsy/prostatectomy) for positive cases; PI-RADS 1-2 screening cases as negative	External validation (ProstateX); compared with radiologist performance (internal reports + external PI-RADS ratings from 4 radiologists)	Internal test set: AUC: 0.89 (DL) vs. 0.89 (radiologists); *p* = 0.88 External test set: AUC: 0.86 (DL) vs. 0.84 (radiologists); *p* = 0.68 Image + radiologist model: AUC: 0.89; *p* < 0.001 vs. radiologists alone Grad-CAM localization: 92% (35/38) internal true-positives; 97% (56/58) external true-positives
Horasan, A.; Güneş, A. (2024) [17]	Detection: Ensemble deep learning model (3D-CNN + ResNet + Inception-v3 with soft voting) for prostate cancer detection in MRI.	bpMRI (T2-weighted, DWI, ADC) Analysis: Lesion-level	*N* = 3458 training/1692 testing (SPIE-AAPM-NCI PROSTATEx dataset) Ref: Transperineal biopsy mapping templates (START criteria)	Hold-out validation on independent test set	Accuracy: 91.3%; Sensitivity: 90.2%; Specificity: 92.1%; Precision: 89.8%; F1-score: 90.0%; AUC: 0.95
Li et al. (2024) [36]	Detection: Unsupervised domain adaptation (UDA) with unified generative model for prostate lesion detection across multisite bpMRI with varying b-values.	bpMRI (T2-weighted, DWI with various b-values, ADC) Analysis: Lesion-level; Case-level	*N* = 5150 patients (14,191 samples) from 9 imaging centers; Test set: 1692 cases (2393 samples) Ref: Expert radiologist-reviewed lesion annotations based on clinical reports	External validation on independent multisite test set; 34 different b-value combinations	Overall (PI-RADS ≥3): Baseline AUC: 0.73 → UDA AUC: 0.79 (*p* < 0.001) Overall (PI-RADS ≥4): Baseline AUC: 0.77 → UDA AUC: 0.80 (*p* < 0.001) Most unfavorable setting (Group 9, PI-RADS ≥3): Baseline AUC: 0.49 → UDA AUC: 0.76 (*p* < 0.001) Most unfavorable setting (Group 9, PI-RADS ≥4): Baseline AUC: 0.50 → UDA AUC: 0.77 (*p* < 0.001)
Li et al. (2024) [41]	Grading: 3D Efficient CapsNet with divide-and-conquer strategy for PCa risk stratification (low/medium/high grade based on Gleason score).	T2-weighted MRI (single modality) Analysis: Lesion-level (3D volumetric)	*N* = 976 (Cancer Imaging Archive, public dataset) Ref: Biopsy-proven Gleason scores (histopathology)	Five-fold cross-validation	Low vs. High: AUC 0.83, F1-score 0.64, Accuracy 0.84 Low + Medium vs. High: AUC 0.79, F1-score 0.75, Accuracy 0.81 Medium vs. High: AUC 0.75, F1-score 0.69, Accuracy 0.71 Low vs. Medium: AUC 0.59, F1-score 0.57, Accuracy 0.60 Final multi-class (divide-and-conquer): Accuracy 0.55, Weighted Cohen’s Kappa 0.41
Sun et al. (2024) [39]	Detection: Cascaded 3D U-Net for csPCa detection and localization.	mpMRI (DWI + ADC maps; T2WI used for preprocessing) Analysis: Lesion-level, Sextant-level, Patient-level	Development: *N* = 2105 (4 hospitals, 2014–2019) External Validation: *N* = 557 (3 hospitals, 2020–2021) Ref: Image-guided biopsy + radical prostatectomy pathology (ISUP grading)	External validation on independent temporal and multi-center cohort	Lesion level: Sensitivity 0.654, Positive Predictive Value (PPV) 0.747 Sextant level: Sensitivity 0.846, Specificity 0.884, Accuracy 0.874 Patient level: Sensitivity 0.943, Specificity 0.776, Accuracy 0.849 csPCa patients accuracy: 0.943 vs. non-csPCa: 0.776 (*p* < 0.001)
Li et al. (2024) [42]	Detection of csPCa aggressiveness; Deep Transfer Learning (DTL) with ResNet50 using 2.5D segmentation (3 consecutive slices as input) vs. 2D model.	bpMRI (T2WI, ADC) Analysis: Lesion-level (manual ROI segmentation on largest lesion layer ± adjacent layers)	*N* = 231 (single center) Ref: Histopathology (Gleason score ≥ 7 = csPCa, <7 = non-csPCa) Split: Training *n* = 185, Test *n* = 46 (stratified random sampling)	Internal validation (single-center, random split)	2.5D Combined Model (Test Set): AUC 0.949; Accuracy 0.884; Sensitivity 0.974; Specificity 0.849 2D Combined Model (Test Set): AUC 0.886 2.5D outperformed 2D in all sequence combinations
Schrader et al. (2024) [32]	Risk assessment for significant prostate cancer (sPC) to avoid unnecessary biopsies; Fully automatic nnUNet-based DL ensemble predicting lesion probability (UNet-probability) and PI-RADS-analogous 5-point scale (UNet-Likert), integrated into risk calculators (RCs).	mpMRI (T2WI, DWI/ADC; radiologists also had DCE) Analysis: Patient-level (whole prostate segmentation, voxel-wise probability maps)	*N* = 1627 consecutive exams (single center, 2014–2021) Ref: Extended systematic + MRI/US-fusion targeted biopsy (ISUP grade ≥ 2 = sPC) Split: Training *n* = 1021 (DL training + RC calibration), Test *n* = 517 (RC evaluation); 834 training/517 test cases without prior PCa for RC analysis	Temporal split validation; internal test set withheld from DL training	UNet-probability alone: AUC 0.89 (95% CI: 0.86–0.92) Newly fitted PI-RADS + UNet-probability RC: AUC 0.93 (95% CI: 0.90–0.95); Brier score 0.10 At 15% risk threshold: 49% biopsies spared (252/517) with Negative Predictive Value (NPV) 94% (vs. 37% spared by PI-RADS ≥4) DL + PI-RADS combination outperformed either alone; UNet-Likert substituted for PI-RADS without performance loss
Talaat et al. (2024) [43]	Detection: Modified ResNet50-based architecture integrating Faster R-CNN with dual optimizers (Adam + Stochastic Gradient Descent (SGD)) for prostate cancer detection; R-mask modification for segmentation.	MRI (histopathological images from prostate cancer dataset) Analysis: Image-level with Mask R-CNN for region segmentation	*N* ≈ 11,000 images (training: ~11,000; test: ~400) Ref: Histopathology (biopsy-confirmed) Source: Kaggle Prostate Cancer Grade Assessment dataset	Internal validation (80% train, 20% test)	Accuracy: 97.40%; Sensitivity: 97.09%; Specificity: 97.56%; Precision: 95.24%
Jin et al. (2024) [29]	Detection & Grading: 3D ResNet18 for prostate cancer detection and Gleason grade prediction using single-modality T2WI.	T2WI (single-modality) Analysis: Prostate-level segmentation (not lesion-level)	Internal: *N* = 497 (195 healthy + 302 PCa); External: *N* = 48; Public challenge: *N* = 91 Ref: Histopathology (biopsy or surgery) Gleason grade groups: 1–5	Multi-center validation (internal + external + public challenge datasets)	PCa Detection: AUC: 0.918 (validation), AUC: 1.000 (training) Gleason Grade Prediction: AUC: 0.854 (validation), 0.776 (external), 0.838 (public challenge); Accuracy: 85.7% (validation), 76.2% (external), 80.6% (public challenge)
Zheng et al. (2024) [44]	Detection: Anatomical-aware PCa detection network (AtPCa-Net) with symmetric-aware architecture and Zonal Loss (ZL) for csPCa detection; 3D UNet-like backbone with nnU-Net structure.	bpMRI (T2WI, ADC, high-B DWI)—DCE excluded Analysis: Lesion-level with patient-level classification	*N* = 652 (Single institution: UCLA) Ref: Whole-mount histopathology (WMHP) after radical prostatectomy; csPCa defined as GS ≥ 7 Includes: 220 patients with PCa (246 lesions) + 432 patients without PCa (negative biopsies)	5-fold cross-validation (internal validation)	Patient-level classification: AUC: 0.880 (95% CI: 0.846–0.914) csPCa detection sensitivity: 67.5% at 0.5 False Positive (FP)/patient; 72.8% at 1 FP/patient; 80.9% at 2.5 FP/patient Compared to nnUNet baseline: AUC improvement from 0.843 to 0.880
Johnson et al. (2025) [40]	Risk stratification & scan tailoring: Multi-task 3D ResNet-50 for simultaneous PI-RADS ≥ 3 and Gleason ≥ 7 classification; real-time workflow integration using Mercure platform.	bpMRI (T2WI, DWI with ADC and b1500) Analysis: Patient-level with real-time inference (14–16 s latency)	Training: *N* = 26,129 studies (20,089 patients, 7 centers, 2015–2023) Prospective test: *N* = 142 (treatment-naive, 2024) Ground truth verified: *N* = 151 (biopsy/prostatectomy/follow-up confirmed) Ref: Prospective: Consensus of 3 radiologists (PI-RADS ≥ 3) Retrospective: Histopathology (Gleason ≥ 7) or long-term follow-up	Prospective validation + Ground truth verified retrospective validation (single institution)	PI-RADS ≥ 3 (prospective): AUC: 0.83 (95% CI: 0.77–0.89); Sensitivity: 93%; Specificity: 54% Gleason ≥ 7 (ground truth): AUC: 0.86 (95% CI: 0.80–0.91); Sensitivity: 93%; Specificity: 62%; PPV: 63% Workflow impact: ~32% of patients (46/142) could avoid mpMRI; ~9 min saved per abbreviated exam

### 3.2. Beyond MRI: AI Applications in Prostate-Specific Membrane Antigen (PSMA) PET/CT and Ultrasound

Beyond MRI, AI is also showing promise in other imaging modalities like PSMA PET/CT and ultrasound. PSMA PET/CT is a powerful tool for PCa staging and recurrence detection [45]. ML models built on its radiomic features have achieved AUCs as high as 0.925 in external validation [46]. Fully automated 3D CNNs can perform whole-body lesion detection with a patient-level sensitivity of 92%, drastically improving the efficiency of nuclear medicine physicians [47]. These results highlight feasibility for automation and quantification; however, clinical impact depends on prospective evaluation, integration into reporting workflows, and cost-effectiveness analyses in representative populations.

In ultrasound, AI primarily serves to enhance biopsy guidance and risk stratification. For example, the AI-guided Ultrasound Strategy for Prostate biopsy (AIUSP) has been shown to improve the detection rates of PCa and csPCa in patients with negative MRI or PI-RADS 3 lesions [48]. Novel techniques combining Stimulated Raman Scattering (SRS) microscopy with CNNs can even perform automated, real-time Gleason grading on unstained tissue with 84.4% accuracy, opening a new frontier for intraoperative diagnostics [49]. These approaches are promising, but their roles relative to MRI pathways (or in MRI-limited settings) require prospective head-to-head comparisons using clinically relevant endpoints (e.g., csPCa detection per biopsy strategy).

Comparative Analysis and Limitations:

AI’s role differs across modalities. In PSMA PET/CT, its primary function is automation and quantification, rapidly and accurately identifying and measuring tumor burden from vast datasets [50]. In ultrasound, AI acts as an “enhancer,” compensating for ultrasound’s lower soft-tissue resolution to improve the precision of biopsy guidance, making it a valuable tool in settings where MRI is unavailable [51].

The limitations of these applications relate to their clinical positioning and technological maturity. PSMA PET/CT is costly, and its AI applications are currently concentrated in academic research and large medical centers [52]. While AI-guided ultrasound is promising, its diagnostic performance requires validation in large-scale, head-to-head comparisons against the gold standard of MRI-guided biopsy to clearly define its place in the diagnostic pathway.

## 4. Artificial Intelligence in Digital Pathology: Automation Meets Histological Complexity

The widespread adoption of WSI is facilitating the digitization of pathology, creating new opportunities for AI integration. In PCa pathology, AI has evolved from basic tissue identification to support automated Gleason grading.

While traditional ML methods, such as those combined with Raman spectroscopy, have shown high accuracy in differentiating tissue types [53], the impact of DL has been substantially more pronounced in the recent literature. DL models trained on WSI can support cancer detection and Gleason grading, and several studies report a high discriminative performance (often AUCs > 0.90 in specific settings) [54,55,56]. However, very high AUC values should be interpreted cautiously because they may reflect (i) highly curated datasets, (ii) patch-level rather than patient-level evaluation, (iii) limited external validation, and/or (iv) optimistic estimates due to leakage if patches or slides from the same patient appear in both training and test sets. In addition, stain/scanner variability and preprocessing choices (including stain normalization) can materially affect generalizability, while label noise arises from inherent inter-observer variability in Gleason grading. Therefore, clinically credible studies should emphasize patient-level external validation, transparent splitting strategies, and report calibration or clinically interpretable operating points for triage use-cases (e.g., “no cancer detected” thresholds with predefined false-negative tolerances). Multiple studies have reported that the grading agreement between AI systems and expert urologic pathologists is very high (Cohen’s Kappa up to 0.872, weighted Kappa up to 0.98) [57,58,59,60,61,62,63,64,65,66,67]. A focused mini-review by Kartasalo et al. summarized that AI-assisted Gleason grading can reach expert-level agreement in controlled settings, but emphasized remaining steps for widespread clinical implementation, including multisite validation, handling domain shift, and quality-control mechanisms (e.g., out-of-distribution detection) [68]. Table 2 summarizes the core data categories extracted from selected key studies of digital pathology for prostate cancer diagnosis.

A landmark achievement in this field is the Food and Drug Administration (FDA) approval of AI products like Paige Prostate Alpha, signifying regulatory acceptance of their safety and efficacy for clinical use [69]. Such regulatory decisions are tied to defined intended uses and do not imply that all pathology AI models or tasks are clinically validated. These systems not only reduce pathologist reading time but also can function as a “second reader,” minimizing missed diagnoses and improving grading consistency [70]. Furthermore, privacy-preserving techniques like federated learning are enabling multicenter model training without sharing sensitive patient data, effectively addressing the “data silo” problem [71,72].

Comparative Analysis and Limitations:

The core value of AI in digital pathology is efficiency, standardization, and quality control. In practice, the most mature clinical applications are triage/second-read support and quantitative assistance within clearly defined workflows; broader tasks (e.g., full case sign-out automation, prediction of molecular subtypes) remain investigational. It is not intended to replace pathologists but to serve as a tireless and highly consistent intelligent assistant. AI excels at high-volume, repetitive screening tasks (e.g., finding small cancer foci across numerous slides), freeing pathologists to focus on complex cases and final diagnostic decisions [73,74,75].

**Table 2:** The core data categories extracted from selected key studies of digital pathology for prostate cancer diagnosis.

Study (Ref.)	Clinical Task	AI Approach & Input	Dataset & Ground Truth	Generalizability (Ext. Val.)	Performance & Implications
Harder et al. (2024) [74]	Virtual Biopsy: Optimize MRI-targeted biopsy approach (number of cores, intercore distance, grading strategy) & Gleason grading.	Method: Pixel-wise segmentation using U-Net++ (decoder) + EfficientNetB1 backbone (encoder) for tumor detection and Gleason pattern (GP3/GP4/GP5) segmentation; fast-track annotation transfer pipeline using DeepLabV3 + ResNet50 for gland segmentation Input: Whole-slide histopathology images (H&E-stained) from radical prostatectomy specimens simulating MRI-targeted biopsies	Train: TCGA cohort *N* = 362 patients (389 WSIs) + University Hospital Cologne *N* = 23 + Case Western University *N* = 30 Virtual biopsy validation: 480 virtual biopsy cores from 114 patients (120 tumors) Independent test: 121 clinically significant MRI-visible tumors from 115 RP patients Ref: Expert GU pathologist manual annotations (pixel-wise); WMHP for final GS	Independent cohort from Wiener Neustadt State Hospital (Austria); validation on MICCAI 2019 Gleason Challenge (222 TMAs)	Tumor detection: Sensitivity 0.99/Specificity 0.90 (aware version); 0.97/0.97 (balanced version) Grading: Quadratic kappa 0.77–0.78 vs. pathologists (non-inferior to inter-pathologist agreement) 65,340 virtual biopsies performed
Kong et al. (2024) [72]	Prostate cancer diagnosis (benign/malignant classification) and Gleason grading (ISUP categories 0–5)	Method: Federated Attention Consistent Learning (FACL) framework based on AttMIL (Attention-based Multiple Instance Learning) with CTransPath feature extractor Input: H&E-stained WSIs cropped into 224 × 224 pixel patches	Train: 19,461 WSIs from 7 centers (Hebei-1/2, Nanchang, DiagSet-B-1/2, PANDA-1/2) Val: 20% internal data from each center Ref: Pathologist annotations (ISUP grading standards)	Diagnosis: Two independent external datasets—DiagSet-A (430 slides) and QHD (765 slides) Grading: Independent dataset from Nanchang hospital	Diagnosis AUC: 0.9718 (vs. single-center average 0.9499) Grading Kappa: 0.8463 (vs. single-center average 0.7379)
Alici-Karaca et al. (2024) [76]	Prostate cancer detection and Gleason grading (8-class/4-class/2-class classification)	Method: Eff4-Attn (EfficientNet-B4 with Efficient Channel Attention (ECA) attention module), replacing original SE blocks with ECA Input: H&E-stained histopathology image patches (380 × 380 pixels), multiple magnifications (5×/10×/20×/40×)	Train/Val/Test: DiagSet-A.1 dataset (238 WSIs, randomly split 8:1:1) 8-class: A (artifact), N (normal), T (tissue), R1–R5 (Gleason grades 1–5) 4-class: A-N-T-R1-R2/R3/R4/R5 2-class: healthy (A-N-T) vs. cancer (R1–R5) Ref: Assessment by two independent pathologists	Random splitting strategy employed to reduce class imbalance effects; no explicitly mentioned completely independent external validation set	Best results at 40× magnification: 2-class accuracy: 96.18% (cancer detection) 4-class accuracy: 94.86% (cancer severity grading) 8-class accuracy: 93.32% (complete Gleason grading)
Kondejkar et al. (2024) [77]	Prostate cancer detection and Gleason grading (9-class patch-level classification: BG, T, N, A, R1–R5)	Method: ResNet-18/34/50 with transfer learning; DeepLabv3 as feature extractor for CNN classifiers Input: 256 × 256 pixel patches from H&E-stained WSIs at multiple magnifications (5×, 10×, 20×, 40×)	Train: DiagSet dataset (~2.6 M tissue patches from 430 fully annotated scans; 4000 images/class curated subset) Val: 5-fold cross-validation Test: 7200 images (stratified split) Ref: Pathologist annotations (9 classes: scan background, tissue background, normal, artifact, Gleason grades 1–5)	Cross-validation across multiple magnification levels; no independent external test set mentioned	ResNet34 Test Accuracy: 0.9999 (40×), 0.9999 (20×), 1.0000 (10×), 0.9993 (5×) ResNet18 Test Accuracy: 0.9977 (40×), 0.9992 (20×), 0.9964 (10×), 0.9921 (5×) ResNet50 Test Accuracy: 0.9957 (40×), 0.9915 (20×), 0.9952 (10×), 0.9981 (5×)
Huang et al. (2024) [78]	Automated Gleason grading of prostate cancer using fast multiphoton microscopy (MPM) with real-time diagnosis capability	Method: SwinIR (image super-resolution) + Swin Transformer (classification); transfer learning from ImageNet-1K Input: Label-free MPM images (unstained tissue); 128 × 128 Low Resolution (LowRes) or 512 × 512 High Resolution (HR); 300 × 300 for classification	Train Super Resolution (SR): 20,272 LowRes-HR image pairs (from 24 TMA spots, augmented to 24,576 pairs) Test SR: 4304 pairs Train Classification: 12,000 images (3500/class: benign, Gleason 3, 4, 5 after augmentation) Test Classification: 2500 images Ref: Pathologist annotations on corresponding H&E-stained sections	Internal validation only; 19 TMA spots (5 tissue microarrays) from single institution	SR Quality: PSNR 24.36 ± 4.38 dB, SSIM 0.9027 ± 0.0130 HR Classification: Accuracy 90.95%, Macro-F1 90.94% SR Classification: Accuracy 89.85%, Macro-F1 89.85% (vs. LowRes: 83.20% accuracy) Speed Improvement: Acquisition time reduced from 7.55 s to 0.73 s per frame (0.24 s LowRes + 0.49 s SR)
Mannas et al. (2024) [79]	Real-time detection of prostate cancer in unprocessed, fresh prostate biopsies using stimulated Raman histology (SRH) with AI interpretation	Method: Inception-ResNet-v2 CNN for patch-level and biopsy-level classification Input: Stimulated Raman histology (SRH) images of fresh, unstained, unlabeled prostate biopsies (label-free, no tissue processing)	Train: 303 biopsies from 100 participants (radical prostatectomy specimens) → 1.75 million patches Val: 4% of total patches (validation set during training) Test: 113 independent biopsies (59 ex vivo, 54 in vivo) from 44 participants Ref: Consensus of 2 genitourinary pathologists on H&E-stained sections (ISUP 2019 grading)	Independent test set with mixed ex vivo and in vivo biopsies; single-center study (NYU Langone Health)	Patch-level accuracy: 98.6% (validation), 99.6% (training) Biopsy-level accuracy: 96.5% (combined ex vivo + in vivo) Sensitivity: 96.3% Specificity: 96.6% AUC: 0.99 Speed: 2–2.75 min per biopsy (full scan); 0.24–0.73 s with 4× accelerated scan Limitation: Cannot assign tumor grades (Gleason grading not performed)
Hossam Magdy Balaha et al. (2024) [66]	Prostate cancer classification (cancer vs. normal) and Gleason grading segmentation (Grade 1–5)	Method: Transfer learning with 8 CNN architectures (ResNet152/152V2, MobileNet/V2/V3Small/V3Large, NASNetMobile/Large) + Aquila optimizer for hyperparameter tuning; U-Net for segmentation Input: H&E-stained histopathology images (PANDA), MRI images (Transverse Plane), ISUP grade-wise images	Train/Val/Test: “PANDA: Resized Train Data (512 × 512)”: segmentation dataset (~11,000 WSIs from Karolinska Institute & Radboud University) “ISUP Grade-wise Prostate Cancer”: 10,616 images (grades 0–5) “Transverse Plane Prostate Dataset”: 1528 MRI images from 64 patients Split: 85% train/val, 15% test for classification; 80% train, 20% test for SR task Ref: Pathologist annotations (ISUP grading for PANDA; significance labels for MRI)	Multi-dataset validation across 3 different data sources (histopathology + MRI); no independent external clinical cohort	Classification Accuracy: ISUP Grade-wise dataset: 88.91% (MobileNet best) Transverse Plane MRI dataset: 100% (MobileNet & ResNet152) Segmentation (U-Net on PANDA): Average accuracy: 98.46% Average AUC: 0.9778 Average Dice: 0.9873 Grade-specific Dice: 0.9761 (G1) to 0.9990 (G5) Optimization: Aquila optimizer improved hyperparameter selection vs. default settings
Yan Gao, Mahsa Vali (2024) [80]	Prostate cancer classification and grading (Gleason grading) from histopathology images.	Method: CNN with hybrid feature extraction Input: Preprocessed pathology images using DWT (Discrete Wavelet Transform) + GLCM (Gray-Level Co-occurrence Matrix) features	Train/Val: PROSTATEx dataset (204 mpMRI scans, 330 lesions for training; 208 lesions for testing) Ref: Pathologist annotation based on Gleason Grade Group (GGG)	Cross-validation with 7:3 train–test split; evaluated on multiple classification tasks (Benign vs. Malignant, Benign vs. Grade 3/4/5, Grade 3 vs. Grades 4&5)	Accuracy: 97.3% (average) Precision: 98% AUC: 0.95 F1-score: 91.05% (macro average)
Xinmi Huo et al. (2024) [65]	AI-assisted Gleason grading for prostate cancer detection and grading from WSIs.	Method: Deep learning classification model (ResNet50, VGG16, NasNet Mobile) with weighted classification layer; Image appearance migration for generalization Input: H&E-stained whole slide images from prostatectomy and biopsy specimens	Train: 131 WSIs (prostatectomy) with 22,148 mm^2^ annotation area (12,630 instances) Val/Test: 56 WSIs (prostatectomy) + 156 biopsy specimens with 2223 mm^2^ annotation area (2852 instances); additional 140 test mpMRIs from PROSTATEx Ref: Multi-pathologist annotation (3 pathologists from NUH, 9 pathologists from 5 Chinese hospitals for validation)	Tested across 6 different scanners (Akoya, Olympus, KFBio, Zeiss Leica, Philips); validated with 5 pathologists from Singapore and China in three-phase clinical study	Annotation-level F1: 0.80 (Akoya), improved to 0.88 with generalization techniques (other scanners) WSI-level Quadratic Weighted Kappa: 0.71 Gleason Pattern Detection F1: 0.73 → 0.88 (with image appearance migration) Time efficiency: 43% reduction in Gleason scoring time Semi-auto annotation efficiency: 2.5× faster than manual annotation

However, the widespread adoption of digital pathology AI faces significant infrastructural and workflow challenges. A primary barrier for many pathology departments is the high upfront investment required for WSI scanners and the associated data storage and management infrastructure. Furthermore, the lack of standardization presents a substantial obstacle; variations in tissue processing, staining protocols, and scanner hardware across different laboratories can lead to image variability, which may degrade the generalization performance of AI models [81]. Finally, successful implementation depends on effective workflow integration. For AI tools to be practical, they must be seamlessly integrated with existing Laboratory Information Systems (LIS), otherwise they risk becoming a burden rather than a benefit to the pathologist’s workflow.

## 5. Artificial Intelligence in Liquid Biopsy: Non-Invasive Detection with Persistent Limitations

Liquid biopsy, which analyzes tumor-derived biomarkers in bodily fluids like blood and urine, offers an attractive non-invasive approach to PCa diagnosis. AI is used to develop multivariable models that integrate weak and heterogeneous biomarker signals (e.g., gene panels, extracellular vesicle markers, metabolite fingerprints) into risk scores.

Using traditional ML, researchers have developed risk prediction models based on urinary gene panels, extracellular vesicle (EV) markers, and metabolite profiles. These models have demonstrated strong performances in distinguishing PCa from benign conditions and in stratifying patients by risk, with AUCs ranging from 0.82 to 0.95 [82,83,84,85,86,87,88]. They are particularly valuable in the PSA “gray zone” (4–10 ng/mL), where they offer diagnostic utility beyond PSA alone. For instance, a model based on urinary EV markers (Epithelial cell adhesion molecule (EpCAM)-CD9) achieved an AUC of 0.712 in this specific population [89]. Similarly, the blood-based EV-MAP model, using EV markers like PSMA, improved the diagnostic AUC from 0.52 to 0.75 [90,91].

DL applications in liquid biopsy are still in an exploratory phase but show promise. For example, combining Raman spectroscopy of urine samples with CNNs offers a novel approach for non-invasive early screening [92].

Comparative Analysis and Limitations:

The primary contribution of AI in liquid biopsy is statistical integration and pattern detection across multianalyte signatures, which can improve discrimination compared with single markers in some cohorts. The greatest clinical value lies in risk stratification and the avoidance of unnecessary invasive procedures if validated prospectively and aligned to prespecified decision thresholds.

The main limitations in this field are related to upstream technological standardization and downstream clinical validation. First, there is a distinct lack of pre-analytical standardization; the entire process, from sample collection and storage to biomarker extraction and detection, lacks uniform protocols, making it difficult to reproduce results across different studies. Furthermore, the detection of weak biological signals presents a significant challenge. The low abundance of biomarkers like ctDNA, especially in localized prostate cancer, places extreme demands on the sensitivity of current detection technologies. Finally, the field suffers from insufficient clinical validation, as most models are currently developed and evaluated solely in retrospective studies. Consequently, large-scale, prospective, multicenter clinical trials are urgently needed to confirm their real-world diagnostic performance and cost-effectiveness.

## 6. Artificial Intelligence in Multimodal Data Integration: Synergy and Complexity

Given the heterogeneity of PCa, multimodal AI models integrating imaging, pathology, genomics, molecular biomarkers, and clinical data are a logical direction for precision diagnosis. In prostate diagnostics, the most clinically actionable near-term multimodal questions are typically “biopsy or defer?” and “targeted vs. systematic strategy?”, especially in biopsy-naïve men and in men with prior negative biopsy and persistent suspicion.

Targeted clinical applications of multimodal AI in PCa diagnosis are increasingly well defined. These models aim to improve risk prediction of csPCa in men with equivocal imaging findings, particularly PI-RADS 3 lesions, thereby guiding biopsy versus active surveillance decisions. In addition, the integration of imaging features with clinical variables, such as PSA, PSA density (PSAD), age, and prostate volume, has been explored to reduce unnecessary biopsies while maintaining high sensitivity. In addition, combining pathology and imaging information may help reduce grading variability and support more consistent risk stratification across institutions. Finally, linking molecular biomarkers (e.g., urine or blood-based panels, methylation signatures, metabolomic profiles) with imaging data may refine individualized probability estimates, particularly when single-modality signals are weak or discordant.

Empirical evidence suggests that such integrative approaches can improve diagnostic performance. By fusing radiomic features, clinical indicators and novel biomarkers, ML models have demonstrated enhanced accuracy in csPCa detection and improved characterization of indeterminate lesions [93,94,95,96,97]. For example, the ClaD nomogram, combining DL features, PI-RADS scores, and clinical parameters, achieved an AUC of 0.81, outperforming any single modality [98]. Similarly, hybrid models that integrate DL-extracted image features with the structured interpretability of traditional ML methods have shown promise in enhancing robustness and clinical utility [99,100].

Comparative Analysis and Limitations:

The conceptual advantage of multimodal AI lies in its ability to generate a holistic, patient-specific profile. Rather than focusing solely on lesion detection, these systems attempt to synthesize anatomical, biological, and clinical signals into a unified risk estimate, thereby moving toward personalized diagnostic and therapeutic decision-making.

However, the complexity of multimodal data integration introduces substantial methodological and practical challenges. Data fusion is inherently difficult due to several factors: (i) heterogeneous data structures, including imaging pixels or volumes, whole-slide pathology tiles, tabular laboratory values, and unstructured text reports; (ii) imperfect correspondence across modalities, such as lesion-level imaging labels linked to patient-level pathological outcomes; (iii) missing data, as not all patients undergo PET imaging, whole-slide imaging, or molecular profiling; (iv) temporal mismatches, where imaging, laboratory biomarkers, and pathology are acquired at different time points; (v) modality-specific noise and batch effects, including scanner and protocol variability in imaging or staining and platform effects in pathology and laboratory assays.

From a methodological perspective, fusion strategies can be broadly categorized into early fusion (feature concatenation at the input level), intermediate fusion (joint representation learning through multimodal encoders), and late fusion (ensemble or stacking approaches combining modality-specific predictors). In practice, late fusion is often easier to validate and recalibrate across sites, whereas intermediate fusion may yield performance gains but can be harder to interpret and maintain under dataset shift. Each approach involves trade-offs in flexibility, interpretability, and robustness. The choice of fusion strategy should be guided by the intended clinical question, available data completeness, and the level of interpretability required for implementation.

Finally, integration may compound black-box effects, further challenging interpretability and clinical trust. As multimodal systems become more sophisticated, transparent reporting, uncertainty quantification, and explainability frameworks will be essential to ensure that performance gains translate into clinically meaningful and ethically responsible deployment. Therefore, multimodal models should report (i) modality contribution analyses, (ii) uncertainty estimates, (iii) performance under missing-modality scenarios that reflect real clinical pathways.

## 7. Discussion

The rapid advancements of AI in PCa diagnosis are promising, but AI translation into routine clinical practice is contingent on overcoming a series of formidable challenges.

### 7.1. Core Challenges and Practical Bottlenecks

The successful translation of AI into routine clinical practice faces several formidable hurdles, starting with the fundamental issue of data governance and heterogeneity. The performance of AI models is intrinsically linked to data quality; however, inconsistent annotation standards, coupled with variability in imaging and pathology protocols, currently hinder the development of robust and generalizable models [101]. Furthermore, data privacy concerns and the prevalent “data silo” effect significantly restrict large-scale collaboration. While privacy-preserving technologies such as federated learning offer promising solutions, the establishment of shared, universal data standards remains a paramount prerequisite for progress [102,103].

A practical limitation of the current evidence base is that many studies remain retrospective, enriched, and heterogeneous in reference standards, making cross-study comparison difficult. For imaging models, the target label may be biopsy-based csPCa, prostatectomy grade group, or radiologist consensus, each carrying different noise and verification bias. Similarly, pathology AI may be trained on slide-level labels but deployed for core-level triage, and liquid-biopsy models may not be validated against standardized pre-analytical pipelines. Therefore, stronger evidence requires prespecified endpoints and reporting: external validation across institutions, calibration and decision-curve analysis (not only discrimination), subgroup performance (e.g., PI-RADS 3, prior negative biopsy), and clinically meaningful operating points reflecting local capacity and risk tolerance. In this respect, confirmatory international studies with paired reader designs (such as PI-CAI) provide a higher-evidence template for evaluating AI not only as an algorithm, but as a clinically relevant diagnostic aid under heterogeneous real-world conditions [27].

Technically, addressing heterogeneity will likely require a combination of harmonization and robustness strategies: scanner- and stain-aware normalization, domain generalization, uncertainty estimation, and continuous performance monitoring after deployment. In MRI, protocol variation (bpMRI vs. mpMRI, b-values, coils) and annotation inconsistency can be partially mitigated by standardized acquisition templates, public benchmarks, and multi-reader adjudication; in digital pathology, color calibration and stain normalization can reduce cross-laboratory drift; and for liquid biopsy, rigorous pre-analytical standard operating procedures are prerequisites before model transportability can be meaningfully assessed.

Beyond data challenges, algorithmic trust and interpretability represent a critical barrier to clinical adoption. The “black box” nature of many advanced deep learning models often leads to hesitation among clinicians, who naturally require an understanding of the rationale behind a diagnostic recommendation. To address this, the integration of Explainable Artificial Intelligence (XAI) is essential. By visualizing the decision-making processes and elucidating associations between biomarkers and clinical factors, XAI enhances transparency, thereby fostering the necessary trust for responsible clinical implementation [30,104].

Even with accurate and interpretable models, clinical workflow integration remains a practical bottleneck. For an AI tool to be viable, it must not disrupt the existing ecosystem but rather interface seamlessly with Hospital Information Systems (HIS), PACS, and LIS. The technology must be fast, user-friendly, and capable of delivering actionable information at the point of care without adding to the clinician’s workload. An AI solution that fails to integrate smoothly into the daily routine is unlikely to succeed, regardless of its diagnostic precision. In pathology, this workflow reality partly explains why clinically deployed systems have focused on high-yield tasks such as cancer detection and second-read triage; independent assessments of such systems (e.g., Paige) underscore that translation depends on operational robustness in addition to headline accuracy [68].

Finally, the deployment of AI necessitates robust regulatory and ethical frameworks. As highlighted by the World Health Organization (WHO), establishing clear accountability structures to address medico-legal liability in the event of AI-driven errors is crucial [105]. Simultaneously, ethical considerations regarding patient autonomy and informed consent must be prioritized. It is imperative that patients fully understand the role and limitations of AI in their care to ensure transparency, trust, and broad societal acceptance.

In addition, real-world adoption depends on safety, fairness, and value. AI systems can fail silently under dataset shift, potentially widening disparities if performance differs across age, ethnicity, MRI access, or center volume. Prospective surveillance plans (periodic re-calibration, drift detection, and human override policies) should be considered part of the “model,” not an afterthought. Economic evaluation is also essential: tools that reduce biopsy rates or pathologist time must demonstrate cost-effectiveness once infrastructure, maintenance, and medicolegal risk are included. Finally, clear accountability models are needed in human. AI teamwork, specifying how AI outputs are presented (probabilities, heatmaps, explanations), how discordant cases are resolved, and how responsibility is shared among clinicians, institutions, and vendors.

### 7.2. “Performance” for Clinical Translation

In the rapidly evolving literature on AI for PCa, performance is most frequently summarized by AUC. However, interpreting these values requires substantial caution. Reported AUCs can vary widely across studies, not only because of algorithmic differences, but also due to heterogeneity in clinical endpoints (e.g., detection of any PCa versus csPCa, Gleason grade group classification, lesion-level versus patient-level analysis), cohort characteristics (screening populations, biopsy-naïve patients, prior-negative cases, and differences in disease prevalence and spectrum), imaging protocols (biparametric versus multiparametric MRI, variations in b-values, field strength, coils, and qualitative versus quantitative assessment strategies), and validation design (random splits, temporal splits, true external multicenter validation, or prospective real-world evaluation).

Such methodological variability makes direct cross-study comparison inherently problematic. Consequently, performance metrics should be interpreted within clearly defined clinical tasks and study designs rather than treated as universally comparable indicators of superiority.

More fundamentally, even a high AUC within a single study does not in itself establish clinical usefulness. For AI systems intended for deployment in PCa diagnosis, performance must be examined across several additional dimensions. First, external validation is critical, particularly in the presence of dataset shift. Performance commonly declines when models are tested on data from different institutions due to variations in scanners, acquisition protocols, staining pipelines, case mix, and prevalence. Therefore, multicenter and temporally separated validation is increasingly important to assess generalizability. Second, calibration must accompany discrimination metrics. A model may achieve a high AUC while providing poorly calibrated probability estimates, limiting its reliability in risk-based decision support such as biopsy triage. Reporting calibration-in-the-large, calibration slope, and, where necessary, recalibration strategies are indispensable when models are used for individualized risk estimation. Third, clinical utility should be quantified in relation to actionable trade-offs. Metrics must be linked to practical consequences, such as the balance between avoided biopsies and missed csPCa, and ideally assessed using decision-curve analysis or net benefit across clinically plausible thresholds. Finally, evaluation integrity must be rigorously protected. Data leakage, such as mixing examinations or image patches from the same patient across training and testing sets, or implicit test-time tuning, can lead to overly optimistic estimates of performance. Strict patient-level data partitioning and transparent reporting standards are therefore essential.

Taken together, performance in translational AI should be understood not as a single summary statistic, but as a multidimensional construct encompassing generalizability, calibration, decision relevance, and methodological rigor. Only within this broader evaluative framework can reported accuracies in imaging and digital pathology be meaningfully interpreted for clinical implementation.

### 7.3. Future Prospects

Despite the challenges, the future of AI in prostate cancer diagnosis is bright, offering transformative opportunities to fundamentally reshape patient care. The ultimate objective is to transition from rigid population-based guidelines to the era of personalized precision medicine. By leveraging multimodal AI models to generate patient-specific risk profiles, clinicians can guide therapeutic selection with unprecedented precision. Crucially, this utility extends beyond initial diagnosis to encompass prognosis and treatment monitoring; emerging models are already demonstrating the ability to predict disease progression, recurrence risk, and response to specific interventions like radiotherapy and hormone therapy, enabling more proactive and customized patient management [106].

Concurrently, AI possesses the potential to democratize diagnostic expertise globally. By embedding the proficiency of expert radiologists and pathologists into accessible clinical support tools, AI can provide critical decision support in underserved areas or to less experienced clinicians, thereby standardizing the quality of care and mitigating healthcare disparities. Furthermore, AI acts as a powerful catalyst for scientific discovery. By mining massive biomedical datasets to discern subtle patterns within genomic, proteomic, and imaging data, algorithms can identify novel biomarkers and uncover new disease mechanisms, generating the hypotheses necessary to accelerate drug development and guide future research directions [107,108].

Several near-term research avenues are particularly promising. Large-scale pre-trained multimodal models that jointly learn from MRI/PET, WSI, and longitudinal clinical data may enable transfer across tasks (detection → grading → prognosis) while reducing the need for large labeled datasets at every site. Uncertainty-aware and risk-calibrated models can better support decisions in gray-zone populations by identifying cases where additional testing (repeat PSA, MRI, targeted biopsy, or liquid biomarkers) is most informative. “Human-in-the-loop” designs should be optimized and tested. Rather than replacing readers, AI can standardize reporting, highlight hard negatives, and quantify tumor burden, with workflows that measure time saved, inter-reader agreement, and downstream management changes. Prospective multicenter trials should evaluate AI as an intervention (e.g., AI-assisted MRI triage or AI-supported Gleason grading), using endpoints such as csPCa detection, biopsies avoided, upgrade rates, and net benefit, coupled with implementation outcomes (acceptability, usability, and integration with PACS/LIS/HIS).

Overall, the field is shifting from “model performance” to “clinical systems engineering,” where data standards, workflow integration, prospective validation, and governance determine whether AI improves patient outcomes. A coordinated agenda (shared benchmarks, transparent reporting, multicenter prospective evaluation, and deployment monitoring) will be decisive for translating promising algorithms into reliable, equitable, and cost-effective PCa diagnostic care.

## 8. Conclusions

Artificial intelligence has shown considerable potential to enhance the diagnosis of prostate cancer, but its clinical maturity varies substantially by domain and task. When evaluating the current landscape, it is crucial to distinguish between near-term clinical applications and ongoing investigational efforts.

In the near term, AI has established a foothold as an assistive tool in specific, well-defined tasks. In digital pathology, AI systems are already being deployed as “second readers” for quality control, demonstrating high reliability in identifying cancer foci and supporting Gleason grading consistency. Similarly, in medical imaging, deep learning-based computer-aided diagnosis (CAD) for MRI is increasingly utilized to standardize prostate volume measurements, assist in lesion segmentation, and provide decision support for equivocal (PI-RADS 3) lesions, thereby helping to optimize biopsy triage.

**Figure 2:**
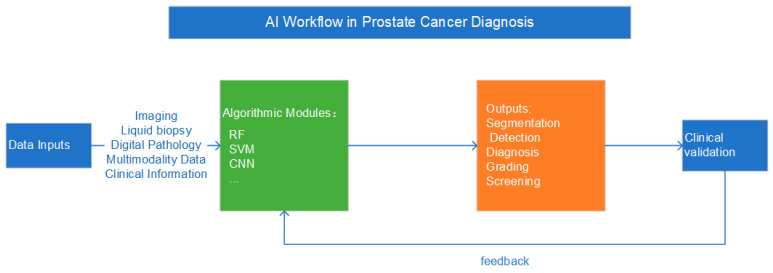
Artificial Intelligence Workflow in Prostate Cancer Diagnosis. This flowchart outlines the workflow of AI technology in the prostate cancer diagnosis process. Multimodal data, including imaging data, liquid biopsy data, digital pathology data, and clinical information, are used as inputs. These inputs are processed through AI models to output results such as prostate cancer stratification, detection, diagnosis, grading, and screening. Clinical validation results are then fed back into the diagnostic process to optimize the model.

Conversely, several applications remain strictly within the research and investigational domain. Fully autonomous diagnostic systems without human oversight are not yet clinically viable. Furthermore, the integration of liquid biomarkers (e.g., ctDNA, exosomes) with AI, and the development of multimodal “fusion” models that simultaneously analyze genomics, radiomics, and clinical data, represent forward-looking research agendas. These complex models promise a future of highly personalized risk stratification but currently lack the pre-analytical standardization and prospective validation required for clinical use. Figure 2 illustrates the specific applications of AI technology in the diagnostic process of PCa.

The path to widespread clinical implementation is paved with practical challenges. Issues of data heterogeneity, model interpretability, clinical workflow integration, and ethical–legal accountability must be systematically addressed. Future research must prioritize the development of robust, generalizable models through large-scale, prospective, multicenter validation rather than relying solely on retrospective performance metrics. Efforts to enhance AI interpretability are critical for building clinical trust. Ultimately, by fostering collaboration between data scientists, clinicians, and regulatory bodies, we can harness the full power of AI to transform prostate cancer care, making it more precise, efficient, and patient-centered.

## Data Availability

This study is a literature review and did not generate new original data. All data analyzed and discussed in this review were derived from published literature cited in the reference list. Information regarding the availability of data from these original studies can be found in their respective publications.

## Supplementary Materials

The following supporting information can be downloaded at: https://www.mdpi.com/article/10.3390/curroncol33030166/s1, Table S1: Characteristics of all 176 included studies.

## Abbreviations

The following abbreviations are used in this manuscript:

ADC	Apparent Diffusion Coefficient
AI	Artificial intelligence
AIUSP	AI-guided Ultrasound Strategy for Prostate biopsy
AUC	Area Under the Curve
bpMRI	Biparametric magnetic resonance imaging
CAD	Computer-aided diagnosis
CI	Confidence Interval
CNN/CNNs	Convolutional Neural Networks
csPCa	Clinically significant prostate cancer
ctDNA	Circulating tumor DNA
DCE	Dynamic contrast-enhanced
DL	Deep learning
DL-CAD	Deep learning-based computer-aided diagnosis
DRE	Digital rectal examination
DSC	Dice Similarity Coefficient
DTL	Deep Transfer Learning
DWI	Diffusion-weighted imaging
DWT	Discrete Wavelet Transform
ECA	Efficient Channel Attention
EpCAM	Epithelial cell adhesion molecule
EV	Extracellular vesicle
FACL	Federated attention consistent learning
FDA	Food and Drug Administration
FP	False Positive
GGG	Gleason Grade Group
GLCM	Gray Level Co-occurrence Matrix
HIS	Hospital Information Systems
HR	High Resolution
IoU	Intersection over Union
ISUP	International Society of Urological Pathology
LIS	Laboratory Information Systems
LowRes	Low Resolution
LogReg	Logistic Regression
miRNAs	microRNAs
ML	Machine learning
MPM	Multiphoton Microscopic
mpMRI	Multiparametric magnetic resonance imaging
MRI	Magnetic resonance imaging
NPV	Negative Predictive Value
PACS	Picture Archiving and Communication Systems
PCa	Prostate cancer
PET/CT	Positron emission tomography/computed tomography
PI-RADS	Prostate Imaging–Reporting and Data System
PPV	Positive Predictive Value
PSA	Prostate-specific antigen
PSMA	Prostate-specific membrane antigen
RC/RCs	Risk Calculators
RF	Random Forest
SGD	Stochastic Gradient Descent
SR	Super Resolution
SRH	Stimulated Raman Histology
SRS	Stimulated Raman Scattering
SVMs	Support Vector Machines
T2WI	T2-weighted imaging
UDA	Unsupervised domain adaptation
WHO	World Health Organization
WMHP	whole-mount histopathology
WSI	Whole-slide imaging
XAI	Explainable Artificial Intelligence
