# Supplementary material for: Current Applications and Future Directions of Artificial Intelligence in Prostate Cancer Diagnosis: A Narrative Review
Source: Curr Oncol. 2026 Mar 13;33(3):166. doi: 10.3390/curroncol33030166 (PMC13025369; doi:10.3390/curroncol33030166)
Supplement: Supplementary file 1 [file curroncol-33-00166-s001.zip › curroncol-4158389-supplementary.pdf]

Supplementary Material

Table S1. Characteristics of all 176 included studies.

Study ID	Country	Study Design	Sample Size	AI / Algorithm Type	Outcomes Extracted
Shao et al., 2022[1]	China	Retrospective; Multicenter (3 hospitals + 1 public dataset)	682 (478 internal + 204 external)	Deep Learning (Topological graph-guided multi-instance network, GMINet)	Accuracy (ACC), AUC, Precision, Recall, F1-score, Free-response ROC (FROC)
Bulten et al., 2021[2]	Netherlands, Multicenter (8 countries)	Prospective observer study; External validation	160 (internal) + 87 (external)	Deep Learning (Gleason grading AI system)	Quadratically weighted Cohen's kappa, Agreement rate, Reading time, Survey results
Pantanowitz et al., 2020[3]	USA, Israel, South Africa	Retrospective; Multicenter (2 centers) + Clinical deployment	2501 (internal) + 1627 (external) + 11,429 (deployment)	Deep Learning (Galen Prostate – Ibex Medical Analytics)	AUC, Sensitivity, Specificity, PPV, NPV, Pearson's r, Bias, Alerts
Jung et al., 2022[4]	South Korea	Independent external validation; User test	593 whole-slide images	Deep Learning (DeepDx Prostate – Deep Bio Inc.)	Sensitivity, Specificity, PPV, NPV, ACC, Kappa, Quadratic-weighted kappa, Spearman's rho, Reading time
Silva et al., 2021[5]	USA	Retrospective; Single-center validation	1876 core biopsies (118 patients)	Machine Learning (Paige Prostate – Paige.AI)	NPV, PPV, Sensitivity, Specificity, F1 score, Discrepancy analysis
Perera et al., 2021[6]	Australia, USA	Retrospective; Multicenter (3 cohorts: PLCO, PCPT, Australian cohort)	10,719 patients	Machine Learning (ResNet18 + GRU with Gaussian process interpolation)	AUC, Sensitivity, Specificity, Calibration
Ström et al	Swede	Population-	8980 biopsy	Deep Learning	AUC, Sensitivity,

al., 2020[7]	n	based; Diagnostic study (STHLM3 cohort + external validation)	cores (training) + 1631 (test) + 330 (external) + 87 (Imagebase)	(Inception V3 ensemble + XGBoost)	Specificity, Cohen's kappa, Correlation (cancer length)
Bhattacharya et al., 2022[8]	USA	Retrospective; Radiology-pathology fusion + external validation	98 (training) + 55 (prostatectomy) + 275 (biopsy) + 39 (normal)	Deep Learning (CorrSigNIA: VGG-16 + CorrNet + modified HED)	Receiver ROC-AUC, Precision-Recall Area Under Curve (PR-AUC), Sensitivity, Specificity, Dice, F1, NPV, PPV
Margineaan et al., 2021[9]	Sweden	Retrospective; Single-center + multi-scanner validation	698 biopsy sections (training) + 37 (test)	Deep Learning (CNN-based Gleason grading tool)	Sensitivity, Specificity, Intraclass Correlation Coefficient (ICC), Cohen's kappa, Agreement Index
Salman et al., 2022[10]	Turkey	Retrospective; Single-center + data augmentation	500 images (training) + 137 (external test)	Deep Learning (Yolo object detection algorithm)	ACC
Winkel et al., 2021[11]	Switzerland, USA, Netherlands, Austria, Russia, South Korea, China, Germany (Multinational)	Retrospective; Multireader multicase (MRMC) study	100 cases (PROSTATEx Challenge)	Deep Learning (Proprietary DL-based CAD system, Siemens Healthineers)	AUC, Sensitivity, Specificity, Reading time, Fleiss κ

Cani et al., 2022[12]	USA	Retrospective; Development and validation	109 patients (73 training, 36 validation)	Machine Learning (Random forest(RF), logistic regression) on RNA-seq data	AUC, Sensitivity, Specificity (for grade group ≥3 prostate cancer)
Hiremath et al., 2021[13]	USA	Retrospective; Multicenter (5 centers)	592 patients (823 lesions)	Deep Learning (AlexNet) + Logistic regression (integrated nomogram ClaD)	AUC, Sensitivity, Specificity, ACC, Biochemical recurrence prediction
Peng et al., 2021[14]	China	Retrospective; Internal and temporal validation	252 patients (135 training, 59 internal validation, 58 temporal validation)	Machine Learning (LR, SR, cDT, CIT, RF, SVM) based on mpMRI radiomics and clinical parameters	AUC, Sensitivity, Specificity, PPV, NPV, ACC
Winkel et al., 2020[15]	Switzerland, USA	Retrospective; Single-center	190 patients (201 lesions)	Machine Learning (GBM, NNet, RF, SVM) using quantitative imaging features (ADC, T2, Ktrans, Kep, Ve)	AUC, Sensitivity, Specificity
Pinckaers et al., 2021[16]	Netherlands, Sweden	Retrospective; Development and validation	5759 biopsies (4712 training, 497 validation, 550 test + 205 external)	Deep Learning (Streaming CNN, ResNet-34)	AUC, Sensitivity, Specificity, Training/Inference time
Yang & Xiao, 2021[17]	China, USA	Retrospective; Multi-center (PANDA challenge dataset)	~11,000 whole-slide images	Deep Learning (MCMS Attention CNN, ResNet50 backbone)	ACC, Quadratic weighted kappa (QWK)
Connell et al., 2021[18]	UK	Retrospective; Single-center (NNUH)	207 patients	Machine Learning (RF, Boruta feature	AUC, Sensitivity, Specificity, OR, Net benefit,

				selection)	Biopsy reduction
Gaudiano et al., 2022[19]	Italy	Retrospective; Single-center	102 patients (117 lesions)	Machine Learning (LASSO + SVM) on ADC radiomics	AUC, Sensitivity, Specificity, PPV, NPV, Informedness
Chen et al., 2021[20]	China	Prospective; Single-center	84 patients (45 BPH, 39 PCa)	Deep Learning (Modified LeNet-5 CNN) on urine Raman spectroscopy	ACC, Sensitivity, Specificity
Dai et al., 2021[21]	China	Retrospective; Single-center	193 patients (112 PCa, 55 BPH, 26 healthy donors)	Machine Learning (Logistic regression)	AUC, Sensitivity, Specificity, PSA gray zone performance
Gentile et al., 2021[22]	Italy	Prospective; Single-center	222 patients (179 training, 43 evaluation)	Deep Learning (Artificial Neural Network)	ACC, Precision, Sensitivity, Specificity, Efficiency
Liu et al., 2021[23]	USA	Retrospective; Single-center	402 patients (239 training, 42 validation, 121 testing)	Deep Learning (3D GLCM + CNN)	AUC, Sensitivity, Specificity
Gravina et al., 2022[24]	Italy	Retrospective; Single-center	109 patients	Machine Learning (RF, SVM, Neural Network, Classification Tree)	ACC, Sensitivity, Specificity, AUC, F1-score
Grosset et al., 2020[25]	Canada	Retrospective; Multicenter (3 centers)	483 patients (272 CHUM, 76 UHN, 135 CHUQc-UL)	Machine Learning (Support Vector Machine, SVM)	ACC, Sensitivity, Specificity, AUC
Kim et al., 2021[26]	South Korea	Retrospective; Single-center	76 urine specimens	Machine Learning (RF, Neural Network)	AUC, Sensitivity, Specificity
Qiu et al., 2022[27]	China	Retrospective; Single-center	321 biopsies (224 training, 87 testing)	Deep Learning (Pyramid Semantic Parsing Network, PSPNet)	ACC, Sensitivity, Specificity, DSC, Kappa, JA, HD, F1-score
Deng et al.,	China	Retrospective; Multicenter (2	262 patients (146 training,	Machine Learning (RF,	AUC, Sensitivity, Specificity

2022[28]		centers)	116 validation)	XGBoost, Logistic Regression, MLP, kNN)	
Huang et al., 2021[29]	USA	Retrospective; Single-center	1000 whole-slide images (589 patients; 838 training, 162 validation)	Deep Learning (U-Net, CNN)	AUC, Weighted κ, Agreement rates, Reading time
Nagpal et al., 2020[30]	USA	Retrospective; Multicenter (3 centers)	752 biopsy specimens	Deep Learning (Neural Architecture Search)	Agreement rate, Sensitivity, Specificity, Mean absolute error (Gleason pattern quantitation)
Aldoj et al., 2019[31]	Germany	Retrospective; Single-center (Charité, Berlin) using public PROSTATEx dataset	200 patients, 318 lesions	Deep Learning (3D CNN)	AUC, Sensitivity, Specificity
Khosravi et al., 2021[32]	USA	Retrospective; Multicenter (1 in-house + 4 public datasets)	400 patients (228 in-house, 172 public)	Deep Learning (Inception-v1, CNN)	AUC, Sensitivity, Specificity, ACC, PPV, NPV, Cohen's kappa
Song et al., 2022[33]	South Korea	Retrospective; Multicenter (2 centers)	12,739 patients in PSA gray zone (2.0–10.0 ng/mL)	Deep Learning (Dense Neural Network, DNN)	AUC, Sensitivity, Specificity, ACC, PPV, NPV
Duran-Lopez et al., 2020[34]	Spain	Retrospective; Single-center (Virgen de Valme Hospital, Seville)	97 whole-slide images (39,677 patches)	Deep Learning (Custom CNN, PROMETEO)	ACC, Specificity, Sensitivity, Precision, F1-score, AUC, Processing time
Yi et al., 2021[35]	China	Retrospective; Multicenter (2 centers)	100 patients (64 training, 36 test)	Machine Learning (RF) on PET radiomics features	AUC, Sensitivity, Specificity, ACC, PPV, NPV
Pellicer-	Spain	Retrospective;	565 patients	Deep Learning	AUC, Sensitivity,

Valero et al., 2022[36]		Multicenter (2 centers: ProstateX + IVO)	(490 training/validation, 75 testing) + ProstateX challenge set	(3D Retina U-Net for detection, segmentation, and Gleason grading)	Specificity, DSC
Arif et al., 2020[37]	Netherlands	Prospective; Single-center	292 patients (low-risk active surveillance cohort)	Deep Learning (3D CNN)	AUC, Sensitivity, Specificity
Bleker et al., 2022[38]	Netherlands	Retrospective; Multicenter (9 centers)	524 lesions (427 patients)	Deep Learning (3D U-Net for prostate segmentation) + Radiomics (XGBoost)	AUC, Sensitivity, Specificity, Reading time
Johnson et al., 2020[39]	USA, China, Sweden, Austria (Multinational)	Retrospective + Prospective; Multicenter (USA, Germany, China)	1010 patients (614 retrospective, 396 prospective)	Machine Learning (RF + Discriminant Analysis on 25-gene panel)	AUC, Sensitivity, Specificity, PPV, NPV, OR
Bulten et al., 2022[40]	Netherlands, Sweden, USA (Multinational; PANDA challenge)	Retrospective; Multicenter (6 sites across EU and US)	12,625 whole-slide images (10,616 development, 545 internal validation, 1,071 external validation)	Deep Learning (Ensemble of CNNs; weakly supervised learning)	AUC, Sensitivity, Specificity, Quadratic weighted κ
da Silva et al., 2021[41]	Brazil, USA	Retrospective; Single-center	600 biopsy part-specimens from 100 patients	Deep Learning (Paige Prostate, CNN-based weakly supervised learning)	Sensitivity, Specificity, PPV, NPV, Reading time reduction
Singhal	India	Retrospective;	6670 whole-	Deep Learning	ACC, QWK,

et al., 2022[42]		Multicenter (3 cohorts)	slide images (3741 training, 425 internal test, 1201 + 1303 external test)	(U-Net with ResNeXt50, ASPP, FPN, multi-task learning)	AUC (cancer detection, low- vs high-grade, GG2 vs GG3)
Steiner et al., 2020[43]	USA	Retrospective; Multicenter (3 centers), multiple-reader multiple-case	240 biopsies from 240 patients	Deep Learning (Google's Gleason grading AI) used as assistive tool	Agreement rate, Sensitivity, Specificity, Reading time, Confidence, Inter-pathologist agreement
Marron-Esquivel et al., 2023[44]	Spain	Retrospective; Multicenter (2 centers: Clinic Hospital + PANDA)	10,696 WSIs (80 local + 10,616 PANDA)	Deep Learning (VGG16, DenseNet121, InceptionV3, custom CNN)	QWK, ACC, Sensitivity, Specificity
Xiang et al., 2023[45]	China	Retrospective; Multicenter (5 centers)	10,616 training slides + 6,174 testing slides	Deep Learning (Weakly supervised learning with graph convolution network, GCN-MIL)	AUC, Sensitivity, Specificity, ACC, Linear/Quadratic kappa
Wang et al., 2023[46]	China	Retrospective; Single-center	729 patients (461 PCa, 268 BPH)	Deep Learning (Multimodal CNN combining serum Raman spectra and clinical features age/PSA)	ACC, AUC
Liu et al., 2024[47]	China	Retrospective; Multicenter (2 centers)	650 patients	Hybrid Model (Radiomics + 3D CNN with CPCB and SE-Net modules) on mp-MRI	ACC, Precision, Recall, F1-score, AUC for GS ≤3+4 vs ≥4+3
Bashkanov et al., 2023[48]	Germany	Retrospective; Single-center	1074 mpMRI scans (1647 lesions, 589 clinically significant	Deep Learning (3D nnU-Net) on biparametric MRI	AUC (patient-level), Partial Area Under the Curve (pAUC) (lesion-level),

			prostate cancer (csPCa))		Sensitivity
Ramirez-Mena et al., 2023[49]	Spain	Retrospective; Multicenter (TCGA + 4 external cohorts)	550 (discovery) + 463 (validation) samples	Machine Learning (RF) based on gene expression data	G-mean, Sensitivity, Specificity, AUC on TCGA; validation AUCs
Harder et al., 2024[50]	Germany, Austria, USA	Retrospective; Multicenter (3 centers)	115 patients (121 MRI-visible tumors)	Deep Learning (U-Net++ segmentation) for tumor detection and Gleason grading + virtual biopsy simulation	Sensitivity, Specificity for tumor detection; quadratic kappa for Gleason grading; AI outperformed 2/3 experts in predicting RP GS
Jaouen et al., 2023[51]	France	Retrospective; Multicenter (4 scanners, 2 institutions for testing)	265 training + 112 pre-test + 158 internal test + 104 external test patients	Machine Learning (Zone-specific ROI-based CAD with logistic regression)	AUC, Sensitivity, Specificity, Comparison with PI-RADSv2
Saha et al., 2024[52]	Netherlands, Norway, Multinational (PI-CAI consortium)	Retrospective; Multicenter (7 sites in Netherlands and Norway)	10,207 MRI examinations (9129 patients)	Deep Learning (Ensemble of 5 top-performing PI-CAI challenge models)	AUC, Sensitivity, Specificity, Comparison with 62 radiologists and standard of care
Li et al., 2024[53]	China	Retrospective; Single-center	133 patients (71 PCa, 62 benign)	Deep Learning (3D-Mask RCNN) on T2WI MRI	DSC, Sensitivity, Specificity, ACC, AUC
Sowmya et al., 2024[54]	India	Retrospective; Single-center (Kaggle dataset)	620 MRI images	Deep Learning (Taylor-AMFSOpt-DAttNNet with HLNLM filtering, HICPIU-Net	ACC, Precision, Recall, F1-score, Specificity, Processing time

				segmentation, Hybrid EfficientNet feature extraction)	
Kong et al., 2024[55]	China, USA	Retrospective; Multicenter (multiple centers in China + public datasets)	19,461 whole-slide images	Federated Learning (Federated Attention Consistent Learning, FACL) with Swin Transformer and AttMIL	AUC, F1-score, ACC, Recall, Kappa
Chandrasekhara et al., 2024[56]	India	Retrospective; Single-center (public dataset)	500 MRI images	Feature extraction (GLCM, SIFT) + Classification (multiclass SVM)	ACC, Sensitivity, Specificity
van den Kroonenberg et al., 2024[57]	Netherlands	Prospective; Multicenter (planned, 5 centers)	438 patients (planned)	Computer-Aided Diagnosis (PCaVision, AI-based on 3D mpUS)	Detection rate, Proportion of biopsies avoided, Diagnostic ACC, Image quality assessment
Zaridis et al., 2024[58]	Greece	Retrospective; Multicenter (public datasets PICAI, Prostate-158)	301 patients (219 + 82)	Deep Learning (ProLesA-Net, multi-channel 3D CNN with multi-scale attention)	Dice score, Hausdorff distance, Average surface distance, Recall, Precision
Sanjid et al., 2024[59]	Bangladesh, China	Retrospective; Multicenter (public datasets ProstateX, Prostate158)	351 patients (193 + 158)	Deep Learning (HUNet, dual-pathway multi-scale hierarchical upsampling network)	Categorical Cross-Entropy (CCE), ACC, DSC, IoU, MHD, Relative Absolute Volume Difference (RAVD), Average Symmetric Surface Distance (ASSD), Average

					Surface Distance (ASD), AUC, Sensitivity, Specificity, False Negative Rate (FNR), False Positive Rate (FPR)
Chen et al., 2024[60]	China	Retrospective; Multicenter (2 centers)	942 patients (432 + 510)	Machine Learning (RF, MLP) based on radiomics features from MRI	ACC, AUC, Sensitivity, Specificity
Kondejkar et al., 2024[61]	USA	Retrospective; Single-center (DiagSet dataset)	430 scans (DiagSet-A.1)	Deep Learning (ResNet-18, ResNet-34, ResNet-50)	ACC, Sensitivity, Specificity, F1-score, Training/Testing time
Ao et al., 2023[62]	China	Prospective; Single-center	104 patients (61 training + validation, 22 external test, 21 clinical simulation)	Deep Learning (Inception-ResNet-v2) on Stimulated Raman Scattering (SRS) microscopy images	ACC, Cohen's kappa, Gleason scoring consistency
Zhang et al., 2023[63]	China	Retrospective; Multicenter (2 centers)	56 patients	Deep Learning (Diagnostic model combining PRIMARY score and SUVmax from PSMA PET/CT)	AUC, Sensitivity, Specificity
Alici-Karaca & Akay, 2024[64]	Turkey	Retrospective; Single-center (DiagSet dataset)	156 WSIs for training, 41 for validation, 41 for testing	Deep Learning (Eff4-Attn: EfficientNet-B4 + Efficient Channel Attention, ECA)	ACC, Precision, Recall, F1-score, Specificity
Song et al., 2023[65]	China, South Korea, Malay	Retrospective; Multicenter (8 centers across Asia)	5037 patients (2231 training, 2806 validation)	Machine Learning (XGBoost, RF, etc.) on	AUC, Sensitivity, Specificity, Unnecessary biopsy

	sia			multimodal health check-up data	reduction, Calibration, Decision curve analysis
Gaudiano et al., 2023[66]	Italy	Retrospective; Single-center	133 patients (155 PI-RADS 3 lesions)	Machine Learning (Support Vector Machine) on ADC radiomic features	AUC, Sensitivity, Specificity, PPV, NPV, Youden Index
El-Melegy et al., 2024[67]	Egypt, Saudi Arabia, USA	Retrospective; Single-center	84 patients	Deep Learning (EfficientNetV2, TabPFN), Ensemble Stacking, and a novel Tab2Visual (tabular-to-image) framework	F1-score, AUC , Brier Score
Yang et al., 2023[68]	South Korea, India, Russia	Retrospective; Single-center (benchmark dataset)	400 MRI samples	Deep Learning (CapsNet, SBiLSTM) optimized by Equilibrium Optimization Algorithm (EOA)	ACC, Sensitivity, Specificity, F1-score
Paproski et al., 2023[69]	Canada	Prospective cohort; Multicenter	215 patients	Machine Learning (XGBoost ensemble) on extracellular vesicle (EV) microflow cytometry data	AUC, sensitivity, unnecessary biopsy reduction
Cai et al., 2024[70]	USA	Retrospective; Multi-site (single academic institution)	5735 examinations (5215 patients)	Deep Learning (3D CNN) trained with patient-level labels; Grad-CAM for localization	AUC, Sensitivity, Specificity, TPR/FPR, Grad-CAM localization success
Qi et al., 2023[71]	China	Retrospective; Single center	383 patients (307 training/valid	Machine Learning (SVM, RF, ADB, GBM)	AUC, Sensitivity, Specificity, ACC

			ation, 76 test)	on radiomics features from transrectal ultrasound videos and mpMRI (T2, ADC)	
Liu et al., 2024[72]	China	Retrospective; Development (TCIA Database), External Validation (Hong Kong Queen Mary Hospital)	820 lesions (718 after resampling) from 75 patients for development; 83 lesions from 8 patients for external validation	Machine Learning (Logistic Regression, RF, SVM) on radiomics features from MRI T2w images	AUC, Sensitivity, Specificity, PPV, NPV
Gavade et al., 2023[73]	India, Norway	Retrospective; Single dataset (I2CVB)	Not specified (I2CVB dataset used, split 90/10 for training/validation)	Deep Learning (U-Net for segmentation, LSTM for classification) on mpMRI	ACC, F1 Score, Precision, Recall (Sensitivity), Specificity, ROC, DSC
Horasan & Gunes, 2024[74]	Turkey	Retrospective; Single dataset (SPIE-AAPM-NCI PROSTATEx)	Not specified (SPIE-AAPM-NCI PROSTATEx dataset used)	Deep Learning Ensemble (3D-CNN, ResNet, Inception-v3) with soft voting on MRI images	ACC, Sensitivity, Specificity, Precision, F1 Score, AUC
Zhang et al., 2023[75]	China	Retrospective; Single center	301 patients (240 training, 61 test)	Machine Learning (ANN, LR, SVM, DT, RF, KNN) on transrectal multimodal ultrasound and PSA-related indicators	AUC, Sensitivity, Specificity, PPV, NPV, F1 Score, Youden Index
Li et al., 2023[76]	China	Retrospective; Single center	166 patients (116 training, 50 test)	Machine Learning (SVM, RF, LDA) on dynamic and static radiomics	AUC, Sensitivity, Specificity, PPV, NPV, ACC, Decision curve analysis

				features from multiparametric MRI (IVIM-DWI, ADC, T2WI)	
Mehmood et al., 2023[77]	Pakistan, Saudi Arabia, Republic of Korea	Retrospective; Single dataset (Cancer Imaging Archive)	221 patients (98 low-grade, 123 high-grade)	Deep Learning / Transfer Learning (EfficientNet) with feature fusion on multi-parametric MRI (T2w, ADC)	ACC, Precision, Recall, F1-score
Zhao et al., 2023	China	Retrospective; Bi-institutional	243 patients (170 training, 73 testing)	Machine Learning (Logistic Regression, XGBoost) on radiomics features from MRI (T2WI, DWI) for PI-RADS 3 lesions	AUC, ACC, Sensitivity, Specificity, Odds Ratio (OR), Decision curve analysis[78]
Hamm et al., 2023[79]	Germany	Retrospective; Single center (with external validation on PROSTATEx)	1224 patients (3260 lesions) for development; 204 patients (330 lesions) for external validation	Explainable Deep Learning (CNN based on VGG) on biparametric MRI (T2WI, DWI)	AUC, Sensitivity, Specificity, ACC, False positives per patient, Reader confidence, Reading time
Huang et al., 2024[80]	China	Experimental; Sample analysis using tissue microarrays (TMAs)	5 TMAs (with 21-22 spots each) from prostate cancer patients	Deep Learning (SwinIR for super-resolution, Swin Transformer for classification) on multiphoton microscopy (MPM) images	Peak Signal-to-Noise Ratio (PSNR), Structural Similarity Index Measure (SSIM), ACC, Macro-Precision, Macro-Recall, Macro-F1
Li et al., 2024[81]	USA	Retrospective; Single dataset (The Cancer Imaging	976 patients (415 low-risk, 445 medium-risk, 116 high-	Deep Learning (3D Efficient Capsule Network) on T2-	AUC, ACC, Precision, Recall, F1-score, Weighted

		Archive)	risk)	weighted MRI	Cohen's Kappa
Li et al., 2024[82]	USA, Germany, Netherlands, Switzerland, Russia, South Korea, China, Austria	Retrospective; Multicenter (9 centers)	5150 patients (14,191 samples) for development; 1692 patients (2393 samples) for testing	Deep Learning (Unsupervised Domain Adaptation with a unified generative model) on biparametric MRI	AUC, FROC, PSNR, Mean Squared Error (MSE), SSIM
Jiang et al., 2023[83]	China	Retrospective; Single center (with external validation on PROSTATEx)	1230 patients for derivation; 169 patients for external validation	Deep Learning (U-Net for segmentation, TrumpetNet for detection) on biparametric MRI (T2WI, DWI, ADC)	AUC, Sensitivity, Specificity, PPV, NPV, ACC, Diagnostic Odds Ratio (DOR)
Liu et al., 2024[84]	China	Retrospective (with prospective testing); Single center	1083 patients for model development (759 training, 324 validation); 147 patients for prospective testing	Machine Learning (LR, XGBoost, RF, Decision Tree, SVM) on clinical and imaging parameters for PI-RADS ≥3 lesions	AUC, Brier score, Sensitivity, Specificity, NPV, Biopsies avoided, Calibration, Decision curve analysis, SHapley Additive exPlanations (SHAP) analysis
Mannas et al., 2024[85]	USA, Canada	Prospective; Single center	416 biopsies (303 training from 100 participants, 113 testing from 44 participants)	Deep Learning (Inception-ResNet-v2 CNN) on Stimulated Raman Histology (SRH) images	AUC, ACC, Sensitivity, Specificity
Zhao et al., 2023[86]	China	Retrospective; Multicenter (7 hospitals)	1861 patients (1216 training, 645 external	Deep Learning (3D CNNs: ResNet3D,	AUC, ACC, Sensitivity, Specificity, DOR

			validation)	DenseNet3D, ShuffleNet3D, MobileNet3D) on biparametric MRI (T2WI, DWI, ADC)	
Roest et al., 2024[87]	Netherlands	Retrospective; Dual center	932 patients (403 for development, 529 for external test)	Multimodal AI (Deep Learning for lesion detection + Machine Learning classifiers) combining MRI features and clinical parameters (PSA, volume, age)	AUC, Information fusion method comparison, Comparison to radiologist performance, Jackknife analysis
Fei et al., 2024[88]	China	Prospective (with external validation); Dual center	427 subjects (367 for model development, 60 for external validation)	Machine Learning on serum metabolic fingerprints (SMFs) from MALDI-TOF mass spectrometry	AUC, ACC, Sensitivity, Specificity, Metabolic pathway analysis, Stage and risk stratification ACC
Sun et al., 2024[89]	China	Retrospective; Multicenter (4 hospitals for development, 3 for external validation)	2105 patients for development; 557 patients for external validation	Deep Learning (3D U-Net) on multiparametric MRI (DWI, ADC)	Sensitivity, Specificity, ACC, DSC, FROC, Bland-Altman analysis
Li et al., 2024[90]	China	Retrospective; Single center	231 patients (185 training, 46 test)	Deep Transfer Learning (ResNet50) with 2.5D segmentation on biparametric MRI (T2WI, ADC)	AUC, ACC, Sensitivity, Specificity, PPV, NPV, F1 score
Schrader et al.,	Germany	Retrospective; Single center	1627 examinations	Deep Learning (nnUNet) on	AUC, Brier score, Calibration

2024[91]			(1021 training, 606 test); 1351 patients without prior PCa for RC analysis (834 training, 517 test)	biparametric MRI; Integration with clinical risk calculators (Radtke, Leeuwen, MRI-ERSPC) and PI-RADS	(Exp/Obs Ratio), Decision curve analysis, Biopsies avoided, NPV
Jafari et al., 2023[92]	Iran	Retrospective; Multicenter (3 centers)	412 patients (200 training, 133 internal test, 47 external test 1, 32 external test 2)	Deep Learning (nnU-Net) for segmentation on [68Ga]Ga-PSMA-11 PET/CT	Patient-level ACC, sensitivity, PPV, NPV; Lesion-level ACC, sensitivity, PPV, F1-score; Voxel-level DSC, PPV, IoU, sensitivity; Correlation of PSMA-TV and TL-PSMA
Yang et al., 2025[93]	China	Retrospective; Dual center (Campus A for training, Campus B for test)	356 patients (241 training, 115 independent test)	End-to-end Radiomics pipeline (6 feature selectors + 5 ML classifiers) on [18F]PSMA-1007 PET/CT with automatic segmentation	AUC, Balanced ACC (bAcc), Calibration, Decision curve analysis, Comparison to clinical model
Marvaso et al., 2024[94]	Italy	Retrospective; Single center	949 patients	Machine Learning (CatBoost gradient-boosted decision trees) on clinical data, radiological scores (PI-RADS, EPE), and whole-prostate radiomics from mpMRI	AUC, Feature importance (SHAP), Mean Absolute Error (MAE), Comparison to naive clinical model, Confusion matrices for staging
Liu et al.,	China	Retrospective; Multicenter (4	750 patients (400 training,	Deep Learning (msVnet) on	DSC, Sensitivity, Specificity, ACC,

2023[95]		centers)	100 validation, 150 internal test, 100 external test)	multiparametric MRI (T2WI, DWI, ADC) for segmentation and CAD (AI-first read, concurrent read)	FPR, FNR, Reader consistency (kappa), Reading time
Gao & Vali, 2025[96]	China, Canada	Experimental; Single dataset (PROSTATEx)	204 patients (with 330 lesions) for model development	Hybrid (GLCM, DWT for feature extraction + CNN for classification) on histopathology images	ACC, Precision, Recall, F1-score, AUC, Specificity, Matthews Correlation Coefficient (MCC)
Wang et al., 2023[97]	China	Prospective; Multicenter (3 centers) Randomized Controlled Trial	400 patients (133 TRUS-SB, 134 mpMRI, 133 AIUSP)	Artificial Intelligence Ultrasound of Prostate (AIUSP) for targeted biopsy	Overall PCa detection rate, csPCa detection rate, Biopsy core positive rate, Number of cores needed to detect one PCa, PI-RADS stratified detection rate
Huang et al., 2024[98]	China	Retrospective; Multicenter (2 centers)	449 patients (342 for model development, 107 for external validation)	Machine Learning (LASSO regression) on clinical data and radiomics features from T2-weighted MRI	OR, C-index, Calibration curve
Yang et al., 2023[99]	China	Retrospective; Multicenter (2 centers for training, 2 independent cohorts for validation)	10,613 WSIs (PANDA) for training; 4626 WSIs (DiagSet-B), 844 WSIs (HEBEI), 50 WSIs (PAIP) for validation	Deep Learning (Intensive-Sampling Multiple Instance Learning, ISMIL) on whole-slide histopathology images	AUC, F1-score, Sensitivity, Specificity, QWK (k_quad), Tumor localization heatmaps
Balaha et al., 2024[100	Egypt	Experimental; Multiple datasets	10,616 images (ISUP), 1,528 images	Hybrid (Deep Transfer Learning with	ACC, Precision, Recall, F1-score, AUC, IoU, Dice

]		(PANDA, ISUP Grade-wise, Transverse Plane Prostate Dataset)	(Transverse Plane), Resized PANDA dataset for segmentation	Aquila Optimizer for classification; U-Net for segmentation)	Coefficient, Sensitivity, Specificity, Cosine Similarity
Zhang et al., 2023[101]	China	Retrospective (with prospective validation); Multicenter (9 hospitals)	4747 patients (4312 for feature selection, 3230 training, 808 internal validation, 519 + 190 prospective validation)	Automated Machine Learning (AutoML) on multimodal data (demographics, labs, ultrasound reports)	AUC, Comparison of AutoML vs. LR/RF/XGBoost, Decision Curve Analysis (DCA), Unnecessary biopsies avoided at 95% sensitivity, NPV, PPV
Wang et al., 2024[102]	China	Retrospective; Single center	54 patients (39 csPCa, 15 BPH/non-csPCa)	Machine Learning (RF) on radiomics features from MRI (T2WI, ADC) and PRKY promoter methylation data	AUC, Sensitivity, Specificity, ACC
Zhao et al., 2024[103]	China	Retrospective; Single center (with temporal validation)	350 patients (191 training, 83 testing, 76 temporal validation)	Machine Learning (XGBoost) on intratumoral and peritumoral radiomics, clinical, radiological (PI-RADS), and metabolic (MRS) features	AUC, ACC, F1 Score, Sensitivity, Specificity, SHAP analysis
Chen et al., 2023[104]	China	Retrospective; Single center	1915 patients (1340 training, 575 test)	Machine Learning (XGBoost) on demographic and serum biochemical parameters	AUC, ACC, Sensitivity, Specificity, DCA, SHAP analysis, Risk thresholds
Talaat et	Egypt,	Experimental;	~11,400 cases	Deep Learning	ACC, Sensitivity,

al., 2024[105]	Saudi Arabia	Single dataset (Prostate Cancer Grade Assessment, PANDA)	(~11,000 training, ~400 test)	(Modified ResNet50 with Faster R-CNN and dual optimizers Adam/SGD) on MRI images	Specificity, Precision
Zheng et al., 2024[106]	USA	Retrospective; Single center	652 patients (220 with PCa, 432 without)	Deep Learning (Anatomical-aware AtPCa-Net) on multi-parametric MRI (T2WI, DWI, ADC)	AUC, Sensitivity at multiple FP/Patient rates (FROC), Comparison to other models (nnUNet, VNet, etc.)
Sun et al., 2023[107]	China	Retrospective; Multicenter (3 hospitals for data, 4 hospitals for readers)	480 patients (180 with csPCa, 300 negative) read by 16 radiologists	Deep Learning-based CAD software as an aid for radiologists	Lesion-level sensitivity, Patient-level sensitivity and specificity, Reading time, Diagnostic confidence
Jin et al., 2024[108]	China	Retrospective; Multicenter (2 centers + public challenge dataset)	597 patients for PCa detection (195 healthy, 402 PCa); 283 patients for Gleason grading	Deep Learning (3D ResNet18) on T2-weighted MRI	AUC, ACC, Recall, F1-score, Comparison to radiologist performance
Yu et al., 2023[109]	China	Retrospective; Multicenter (4 centers)	1540 patients (707 from center 1, 532 from center 2, 163 from PROSTATEx, 138 from Prostate158)	Deep Learning (UNet-Seg for detection, 3D-ResNet with UDM for PI-RADS scoring)	Dice score, Sensitivity, Quadratic-weighted Kappa, AUC, Agreement with subspecialists, Comparison to general radiologists
Bao et al., 2023[110	China	Retrospective; Dual center	1442 patients (903 from center 1, 539	Hybrid (Stack-ensemble deep learning +	Macro-AUC, ACC, F1-score, Upgrade/downg

]			from center 2)	Machine Learning) on clinical data and MRI radiomics	rade rates, C-index for Biochemical Recurrence (BCR) prediction, Comparison to biopsy
Shi et al., 2023[111]	China	Retrospective; Single center	201 subjects (105 healthy controls, 96 prostate cancer patients)	Machine Learning (OPLS-DA, PLS-DA, SVM, RF, Tree, kNN, NN) on urinary metabolic fingerprint data from mass spectrometry	AUC, ACC, Confusion Matrix
Tolkach et al., 2023[112]	Germany, Austria, Netherlands, Israel, Japan, Vietnam, Russia, USA	Retrospective; Multicenter (5 centers)	5922 H&E sections (7473 biopsy cores) from 423 patients for detection; 227 and 159 tumor-bearing cores for grading	Deep Learning (clinical-grade classifier) on whole-slide histopathology images	Sensitivity, Specificity, NPV, False positives, False negatives, QWK for Gleason grading
Huo et al., 2024[113]	Singapore, China	Retrospective; Single center (with multi-scanner and multi-pathologist validation)	187 prostatectomy specimens and 156 biopsy specimens for annotation; 39 slides for PAI experiment; 5 pathologists for clinical validation	Deep Learning (ResNet50, VGG16, NasNet Mobile) with AIMagQC, AIHistoClouds, and Pathologist-AI Interaction (PAI)	F1 score, QWK, Sensitivity, Specificity, NPV, Reading time, Annotation time improvement
Alzate-Grisales	Spain, Colom	Retrospective; Dual dataset	1649 images (1014 training,	Deep Learning (SAM-UNETR,	Dice Score, IoU, AUC

et al., 2023[114]	bia, Chile	(Prostate158 and PI-CAI Challenge)	318 validation, 317 test)	UNET, UNETR, SwinUNETR) on multi-parametric MRI (T2w, ADC, DWI) for lesion segmentation	
Pan et al., 2024 [115]	China	Retrospective; Single-center	108 patients (45 PCa, 63 BPH)	Machine Learning (Support Vector Machine, Random Forest, Boost Tree, Decision Tree) on prostate fluid metabolic fingerprint (PSF-MF) data from FeNPALDI-MS and clinical feature (PSA)	AUC, Sensitivity, Specificity, Metabolic pathway analysis, Feature selection
Martelin et al., 2024[116]	France	Retrospective; Analysis of PLCO trial data	34,224 patients (22,188 training, 12,036 validation)	Machine Learning (Gradient Boosting) on PSA, DRE, and personal/demographic data	AUC, PPV, Risk stratification (high, intermediate, low)
Jia et al., 2025[117]	Sweden, United Kingdom, Finland, Norway, Denmark, Australia, USA	Retrospective; Multicenter (4 centers)	3651 biopsy cores for training (STHLM3); 1161 cores for external testing (Karolinska, Aarhus, Stavanger)	Deep Learning (Fully supervised InceptionV3 with gradient-boosted trees; Weakly supervised foundation model UNI) with physical color calibration	Cohen's kappa, Sensitivity, Specificity, Linear correlation for cancer length, ROC, Calibration curves
Smelik et al., 2025[118	Sweden, China,	Retrospective; Multi-cohort analysis	>2000 patients across multiple	Machine Learning (Ridge regression) on	AUC, Correlation, RMSE, R^2,

]	United Kingdom		datasets (ST, scRNA-seq, bulk RNA, proteomics)	spatial transcriptomics, proteomics, and clinical data	Feature importance (SHAP), Survival analysis (Cox proportional hazards)
Du et al., 2025[119]	Sweden, Switzerland	Microsimulation model based on Swedish population data	10 million simulated individuals	AI-assisted pathology workflow (AI for pre-screening, pathologist for positive cores) modeled within a microsimulation framework	PSA tests, Biopsy procedures, PCa incidence, Overdiagnosis, PCa deaths, Quality-Adjusted Life Years (QALYs), Costs, Incremental Cost-Effectiveness Ratio (ICER), Reduction in pathologist-evaluated cores
Twilt et al., 2025[120]	Netherlands, United Kingdom, Germany, Italy, USA, Norway	Prospective observer study; Multicenter (4 centers for data, 53 centers for readers)	360 MRI examinations read by 61 radiologists	Deep Learning-based AI system (ensemble of top 5 PI-CAI algorithms) as concurrent reader aid	AUC, Sensitivity, Specificity, Comparison of AI-assisted vs. unassisted reading, Subgroup analysis by reader expertise
Choi et al., 2025[121]	South Korea	Prospective; Single center (with blinded validation)	102 patients (after exclusions, 97 used for analysis)	Explainable AI (XAI) with logistic regression and SHAP on data from a dual-gate FET biosensor	AUC, Sensitivity, Specificity, ACC, Feature importance (SHAP), Comparison to PI-RADS
Sun et al., 2025[122]	China	Retrospective; Dual center	900 patients (356 csPCa, 544 non-csPCa)	Deep Learning-based CAD software as an aid for	Lesion-level sensitivity, Sextant-level AUC, Patient-

				radiologists of varying experience levels	level AUC, Reading time, Diagnostic confidence, Inter-reader agreement (Fleiss κ)
Padhani & Papanikolaou, 2025[123]	United Kingdom, Portugal	Special Report / Review; Opinion piece	Not applicable	Conceptual framework for AI-integrated workflows (decision support, safety net, triage, filtering, autonomous)	Qualitative analysis of workflow types, positive/negative human-AI interactions, performance requirements (rule-in/rule-out)
Zhong et al., 2025[124]	China	Retrospective (multi-cohort) with prospective clinical validation; Single center	1096 patients from public databases for model development; 72 patients (42 PCa, 30 BPH) for clinical validation	Machine Learning (113 combinatorial models with 12 algorithms: Lasso, Ridge, Enet, Stepglm, SVM, glmBoost, LDA, plsRglm, RandomForest, GBM, XGBoost, NaiveBayes) on transcriptomic data	AUC, Model performance comparison, Gene expression validation in cell lines and plasma, Logistic regression
Zaheer et al., 2025[125]	Saudi Arabia, Pakistan	Retrospective; Single dataset (SICAPv2)	10,000 whole slide images (70% training, 15% validation, 15% test)	Deep Learning (Transformer-Attention Hybrid U-Net, TAH U-Net) on histopathology images	Dice Score, IoU, Precision, Recall, F1-score, Explainability (Grad-CAM, LIME, PDP)
Zhou et al., 2025[126]	China	Retrospective; Single center	199 patients (139 training, 60 validation)	Machine Learning (RF, XGBoost, Extra Trees) on radiomics features from multimodal MRI	AUC, ACC, Recall, Precision, F1-score, SHAP analysis

				(ADC, T2) and peritumoral regions	
Zhang et al., 2025[127]	USA, China	Retrospective; Single center	199 patients (106 BPH-Only, 93 BPH-PCa)	Deep Learning (3D conditional GAN, ProZonaNet) for prostate zonal segmentation on T2-weighted MRI	DSC, IoU, PPV, Concordance Correlation Coefficient (CCC), OR, AUC, SHAP analysis
Zhang et al., 2025[128]	China	Retrospective; Single center	109 patients (52 PCa, 57 BPH)	Machine Learning (RF) on radiomics features from mpMRI (T2WI, ADC) and clinical data (TAP, PSA, age, volume)	AUC, Sensitivity, Specificity, ACC, Precision, Recall, F1-score
Zarei et al., 2025[129]	Iran	Experimental; Single dataset (clinical dataset from Iran)	1000 histopathology images (70% training, 30% test)	Deep Learning (developed manifold-based deep learning) on histopathology images with Tile and Grad-CAM features	ACC, AUC
Telecan et al., 2025[130]	Romania	Retrospective; Single-center	154 patients, 201 lesions	Machine Learning (RF, SVM, Logistic Regression) on T2WI radiomics + clinical features	ACC, Sensitivity, Specificity, PPV, NPV, FPR, FNR, False Discovery Rate (FDR), Feature importance ranking
Stojadinovic et al., 2025[131]	Serbia	Retrospective; Single-center	729 patients (234 PCa, 120 intermediate-risk)	Machine Learning (Generalized Linear Model, GLM) on pre-biopsy clinical features	AUC, ACC, Sensitivity, Specificity, Calibration, Decision curve analysis, Feature importance

Srivastava et al., 2025[132]	India	Retrospective; Dataset-derived (PANDA challenge subset)	11,684 training, 2,854 testing, 2,939 validation patches	Deep Learning (Parallel Branch Network with Efficient Residual and Attention Blocks) on histopathology images	ACC, Precision, Recall, F1-score, Computational complexity (Parameters, Floating Point Operations (FLOPs), Test time)
Yuan et al., 2025[133]	Australia, China	Retrospective; Multi-center (PI-CAI challenge dataset)	10,000+ bpMRI exams	Deep Learning (Zonal-aware Self-supervised Mesh Network) on bpMRI	Average Precision (AP) score, AUC, FROC, Ablation study results
Xu et al., 2025[134]	China	Retrospective; Single-center	106 patients, 274 ROI sets	Deep Learning (ResNet50 + Multi-head Attention) on mpMRI (T2W, DCE, DWI)	AUC, PR-AUC, ACC, F1 Score, Confusion Matrix, Per-modality performance
Wu et al., 2025[135]	China	Retrospective; Single-center	508 patients (PSA 4–10 ng/mL)	Machine Learning (SVM, RF, ADB, GBM) on TRUS video radiomics	AUC, ACC, Sensitivity, Specificity, PPV, NPV, F1-score
Qiu et al., 2025[136]	China	Retrospective; Dataset-derived (PANDA and ARVANITI)	PANDA: approx. 10,000+ WSIs; ARVANITI: 167 cores (tissue microarrays)	Deep Learning (Channel and Pixel Attention-based Encoder + Transformer/CNN Dual-branch Decoder) on histopathology images with stain deconvolution	ACC, Cohen's Quadratic Kappa, Binary classification performance, Ablation study (CPAM module, decoder, stain channel combinations), Feature visualization (Grad-CAM)
Saitta et al., 2025[137]	Italy	Retrospective; Single-center	1422 patients (691 csPCa, 731 non-csPCa)	Machine Learning (Multivariable Logistic Regression, Boruta feature	AUC, Sensitivity, Specificity, NPV, OR for predictors, Decision tree structure

				selection, CART decision tree) on clinical + imaging (microUS + MRI) data	
Sadeghi et al., 2025[138]	Iran	Retrospective; Single-center	15 patients, 80 lesions (56 csPCa)	Machine Learning (SVM, RF, Logistic Regression, K-Nearest Neighbors) on mpMRI + 68Ga-PSMA PET/CT radiomics	AUC, Sensitivity, Specificity, ACC, Precision, Recall, Net Reclassification Index (NRI)
Rotteveel et al., 2025[139]	USA, Netherlands	Retrospective; Multicenter (4 centers)	365 urine samples (125 control, 133 low-risk, 107 high-risk)	Deep Learning (CNN, ResNet18) + Domain Adversarial Learning on GC-MS volatilome data	ACC, Recall, Precision, F1-score, Bias reduction analysis (PCA clustering)
Rodrigues et al., 2025[140]	Portugal, Multicenter (12 centers across Europe)	Retrospective development + Prospective validation; Multicenter (12 centers, 9 countries)	7157 training, 200 retrospective validation, 1629 prospective validation	Machine Learning (LightGBM, CatBoost) on bpMRI radiomics (whole-gland) + clinical features (age, PSA, PI-RADS, lesion location)	AUC, Specificity, Unnecessary biopsy reduction, Sensitivity analysis, SHAP analysis, Fairness analysis, Decision curve analysis
Nissler et al., 2025[141]	Germany	Retrospective; Single-center, fully crossed multireader multicase	105 patients (52 controls, 53 with PCa)	Sequential deep learning network (aniso-3D U-Net + 3D CNN) for lesion segmentation and classification	AUC, Sensitivity, Specificity, pAUC, Likelihood ratios
Mulligi et al., 2025[142	Sweden, Norwa	Retrospective; Multicenter (international)	>45,000 slides across multiple	Deep neural networks for Gleason grading,	QWK, Linear Weighted Kappa (LWK), AUC,

]	y, Finland, Denmark, Poland, Switzerland, Australia, Japan	, protocol-based development and validation	cohorts (STHLM3, PANDA, etc.)	cribriform cancer, PNI, and cancer extent	Sensitivity, Specificity, RMSE, Cohen's kappa
Prabhu et al., 2025[143]	India	Retrospective; Deep learning model development and validation on public and private datasets	Prostate Gleason dataset (~70,000 patches), KMC Kidney dataset (4,441 patches)	Custom CNN (ProsGradNet) with Context-Guided Shared Channel Residual (CGSCR) blocks	ACC, Precision, Recall, F1-score, Confusion matrix, FLOPS, Parameters
Pausch et al., 2025[144]	Switzerland	Prospective; Single-center	123 biopsy-naive patients	Deep learning-assisted MRI reconstruction (Deep Resolve) for T2w and DWI sequences	Sensitivity, Specificity, PPV, NPV, Diagnostic ACC, mPI-QUAL score, Inter-reader agreement (Cohen's κ)
Oka et al., 2025[145]	Japan	Retrospective; Single-center, post-prostatectomy pathology correlation	254 patients (127 with GGG ≥4, 127 with GGG ≤3)	3D Deep Convolutional Neural Network (3DCNN) on T2w and ADC sequences	AUC, Sensitivity, Specificity
Lou et al., 2025[146]	China	Retrospective; Multicenter (4 centers)	815 men (development: 552, internal test: 93, external test 1: 96, external test 2: 74)	Inflated 3D ConvNet (I3D) on TRUS video clips	AUC, Sensitivity, Specificity, ACC, F1-score, Kappa
Mitura et al., 2025[147	Poland	Prospective; Single-center	93 subjects (53 PCa, 40 healthy)	PCA, kNN, RF, SVM on Raman spectra of serum	ACC, Sensitivity, Specificity, AUC, PCA loading

]				and urine	plots
Miao et al., 2025[148]	China	Retrospective; Single-center	341 patients (119 ISUP <3, 222 ISUP ≥3)	Few-shot deep learning (MSNet) with multimodal imaging (PET, CT, DWI, T2WI, ADC)	AUC, ACC, Sensitivity, Specificity, F1-score, Calibration, DCA
Luo et al., 2025[149]	China	Retrospective; Single-center	131 patients (62 csPCa, 69 non-csPCa)	3D U-Net on mpMRI + logistic regression with PHI, PSA, age, PIRADS	AUC, R^2, Sensitivity, Specificity, DCA, Nomogram
Lu et al., 2025[150]	China	Retrospective; Single-center	1,195 TRUS images from 184 patients (74 benign, 110 malignant)	Deep Augmented Metric Learning (DAML) with SDMS, LIAS, PARL	Precision, Recall, F1-score, t-SNE visualization
Li et al., 2025[151]	China	Retrospective; Single-center	211 patients (148 training, 63 testing)	Logistic Regression; Radiomics feature extraction from intralesional and perilesional ROIs on bp-MRI	AUC, Sensitivity, Specificity, ACC, ROC curves, DCA, Calibration curves
Liu et al., 2025[152]	China	Retrospective; Single-center	542 patients (434 training, 108 testing)	nnU-Net for 3D prostate segmentation; PyRadiomics feature extraction; 113 ML models including RF, Lasso, SVM, XGBoost, etc.	AUC, Dice coefficient, Relative Volume Error (RVE), 95% Hausdorff Distance (HD95), ROC, DCA
Liu et al., 2025[153]	China	Retrospective; Internal and external validation (ProstateX + Huashan)	292 patients (200 internal, 92 external)	CCT-Unet for zonal segmentation; Radiomics + LASSO + SHAP; LDA, LR, SVM	AUC, Sensitivity, Specificity, ACC, Youden Index, SHAP-based feature contribution

				classifiers	
Liu et al., 2025[154]	Australia	Prospective; Multicenter (Epworth hospitals)	60 patients	PSMA PET/CT + mpMRI; PRIMARY score; PSMA-targeted biopsy	Detection of MRI-occult lesions, csPCa detection rate, treatment recommendation change, SUVmax, PRIMARY score distribution
Ling et al., 2025[155]	China	Systematic review and meta-analysis	16 studies (17,316 patients)	Various ML models including LR, RF, SVM, DL, hybrid models; imaging + clinical + pathological data	Pooled AUC, subgroup AUC by model type, data type, time interval; heterogeneity (I^2), funnel plot
Kim et al., 2025[156]	USA	Prospective cohort; Single-center	241 patients (217 included after exclusions)	Machine Learning (RF, SVM, XGBoost) on DBSI metrics; model named DHI (Diffusion Histology Imaging)	AUC, Sensitivity, Specificity, Biopsy reduction, NPV, OR, ROC, DCA
Li et al., 2025[157]	China	Retrospective; Dual-center (Shanghai General Hospital, West China Hospital)	548 patients (288 internal, 260 external)	Machine Learning (LightGBM, RF, XGBoost, SVM, etc.) on clinical and biopsy data	AUC, ACC, Sensitivity, Specificity, Youden Index, F1 Score, Calibration, DCA, SHAP interpretation, Online tool
Lee et al., 2025[158]	South Korea	Prospective; Bicenter (Seoul St. Mary's Hospital, Eunpyeong St. Mary's Hospital)	205 men with 259 lesions	Deep Learning (syngo.via Prostate MR, Siemens Healthineers) on bp-MRI	AUC, Sensitivity, Specificity, PPV, NPV, Per-lesion and per-patient analysis, Subgroup analysis by lesion volume

					and zone
Lai et al., 2025[159]	China	Retrospective; Mixed cohort (public database)	8,741 participants (PCa, OCa, BPD, HP)	Machine Learning (AdaBoost, XGBoost, SVM, RF, LR, KNN) on serum miRNA (miR-1290, miR-6777-5p, miR-1343-3p, miR-6836-3p)	AUC, Sensitivity, Specificity, ACC, PPV, NPV, DCA, PCA, Subgroup analysis by age and stage
Gupta et al., 2025[160]	India, Canada, USA	Systematic review; 29 studies (2019–2023)	17,954 participants	Deep Learning (CNN, U-Net, ResNet, VGG, etc.) on mpMRI (T2WI, DWI, ADC)	AUC, Sensitivity, Specificity, ACC, PPV, NPV, PI-RADS comparison, Detection rates, Image quality (Signal-to-Noise Ratio (SNR), Contrast-to-Noise Ratio (CNR)), Acquisition time reduction
Giganti et al., 2025[161]	UK	Retrospective; Multicenter (6 UK hospitals) + PROSTATEx dataset	1,045 patients (793 training, 252 validation)	Deep Learning (Pi, CE-marked DL-CAD medical device) on mpMRI	AUC, Sensitivity, Specificity, PPV, Non-inferiority analysis, Per-site AUC, fROC analysis
Johnson et al., 2025[162]	USA	Retrospective and prospective; Single-center (NYU)	26,129 training studies; 151 retrospective + 142 prospective test patients	Deep Learning (3D ResNet-50, multitask learning) on bpMRI (T2WI, DWI, ADC, b1500)	AUC, Sensitivity, Specificity, Real-time inference latency, Confusion matrix, Potential biopsy reduction
Jiang et al., 2025[163]	China	Retrospective; Dual-center (Zhejiang University, Jilin Cancer Hospital)	222 patients (201 internal, 21 external validation)	Deep Transfer Learning (ResNet-50) + Radiomics on 18F-PSMA PET/CT;	AUC, Sensitivity, Specificity, PPV, NPV, ACC, SHAP interpretation, Nomogram,

				Multimodal fusion (imaging + clinical)	DCA
Harmanani et al., 2025[164]	Canada	Retrospective; Multicenter (5 centers)	693 patients (6607 biopsy cores)	Deep Learning (Self-supervised VICReg, Transformer-based MIL, RUSBoost ensembles) on micro-ultrasound	AUC, Balanced ACC, Sensitivity, Specificity, Expected Calibration Error, Brier score, Uncertainty-based rejection analysis, Heatmap visualization
Hamid et al., 2025[165]	Indonesia	Retrospective; Single-center (Universitas Indonesia)	529 whole-slide images → 26,418 image patches	Deep Learning (CNN: Xception, EfficientNetV2Bo; Transformer: ViT, DeiT; Hybrid: ConvNeXt-Tiny) on histopathology H&E slides	AUC, Sensitivity, Specificity, PPV, NPV, ACC, F1-score, Confusion matrices, Inference time
Deniffel et al., 2025[166]	Canada, Switzerland, Germany	Retrospective; Two-center (Sunnybrook Health Sciences Centre, University Health Network, Toronto)	278 patients (202 training, 76 validation)	Local clinical risk model (elastic net regression) using age, log-prostate volume, log-PSAD, prior negative biopsy; compared to PSA density, normalized ADC, lesion volume, MRI-ERSPC, Radtke model	AUC, Decision curve analysis (net benefit), Net biopsy reduction per 1000 men, Comparison of strategies at risk thresholds
Geng et al., 2025[167]	China	Retrospective; Two-center (Radiology and Nuclear Medicine)	539 patients (276 training, 185 test, 78 validation)	Multi-task deep learning (3DUnet-based) on plain abdominal CT;	AUC, ACC, Sensitivity, Specificity, Calibration curves, Hosmer–

				compared with single-task ResNet18 and radiomics model; nomogram incorporating PSAD and age	Lemeshow test, Kappa consistency, Grad-CAM visualization
Du et al., 2025[168]	China	Retrospective; Single-center (Dongyang Hospital of Wenzhou Medical University)	928 patients (742 training/validation, 186 test)	Machine learning (LightGBM, Logistic Regression, Decision Tree, SVM, RF, XGBoost) on PSA indicators + TRUS video radiomics (851 features, reduced to 7 via LASSO)	AUC, ACC, Sensitivity, Specificity, PPV, NPV, F1-score, SHAP, Calibration curves, Decision curve analysis, Brier score
Du et al., 2025[169]	Sweden	Microsimulation model (based on STHLM3 data, Swedish population)	10 million simulated individuals	AI-assisted pathology workflow (AI screens biopsy cores, pathologist reviews only AI-positive cores)	Biopsy core reduction, PSA tests, biopsy procedures, PCa incidence/mortality, overdiagnosis, QALYs, costs, ICER, probabilistic sensitivity analysis
Dimitriadis et al., 2025[170]	Greece, Spain, Italy, Portugal (multinational)	Retrospective + prospective; Multicenter (13 centers, ProstateNET)	6458 retrospective, 436 prospective patients	Deep learning (Multi-Encoder Cross-Attention Fusion) on bpMRI (T2w, DWI, ADC), with clinical variables (age, PSA, PSAD)	AUC, Sensitivity, Specificity, PPV, NPV, Fairness analysis (by PSA, age, site, field strength, manufacturer), DeLong test
Zaridis et al.,	Greece,	Retrospective; Multicenter	4816 patients (3656 training,	Automated Machine	AUC, Sensitivity, Specificity, ACC,

2025[171]	Portugal, Italy, Spain (multinational, pan-European)	(12 clinical centers across Europe, ProstateNET)	1162 prospective validation)	Learning (AutoML) framework (Simplatab) with XGBoost, RF, SVM, Logistic Regression, etc., on radiomics features from bpMRI	F1-score, Balanced ACC, SHAP analysis, Bias detection metrics, Model vulnerability detection, Calibration
Antonutti et al., 2025[172]	Italy, Austria	Retrospective; Single-center (PI-CAI dataset)	1500 patients (1275 labeled, 225 unlabeled)	Deep Learning (UNet++ for segmentation, EfficientNet for classification) with pseudo-labeling and multi-task fusion on bpMRI slices	AUC, AP, Inference speed, VRAM(Video Random Access Memory) usage, Ablation study results
Debs et al., 2025[173]	France (multinational training data from 9 countries across 3 continents)	Retrospective; Multicenter (9 providers across North/South America, Europe, Asia for training; PROSTATEx for testing)	4381 training, 328 testing (PROSTATEx)	Deep Learning (3D nnU-Net) on bpMRI; compared to non-expert radiologists using mpMRI	AUC, Lesion-level sensitivity), Average Precision, Sensitivity by lesion volume and Gleason Grade Group, Comparison with radiologists (True Positive Rate (TPR) at equivalent FPR)
Chen et al., 2025[174]	China	Prospective cohort; Single-center (Tongji Hospital)	107 sample points from 22 volunteers (12 for training, 10 for external validation)	Deep Learning with Supervised Contrastive Loss (CNN-based, SC model, SCL-adjust model) on photoacoustic spectroscopy data	ACC, Precision, Recall, Specificity, AUC, Silhouette score, Noise robustness analysis, Model transfer performance
Arafa et al., 2025[175	Saudi Arabia	Retrospective; Multicenter (2 hospitals in	969 patients (577 biopsy-negative, 95	Machine Learning (RF, Extra Tree,	ACC, Precision, Recall, F1-score, AUC, External

]		Riyadh)	NCSPCa, 297 CSPCa)	Decision Tree, XGBoost, Gradient Boosting, Linear Regression) on clinical parameters (age, PSA, PSAD, PV, mpMRI PI-RADS)	validation performance, Subgroup analysis by PSA cutoff (≤10 vs >10 ng/mL)
Ayyildiz et al., 2025[176]	Turkey, USA	Retrospective; Single-center (Istanbul University, Istanbul Faculty of Medicine)	77 patients (30 clinically insignificant prostate cancer (ciPCa), 47 csPCa)	Machine Learning (SVM, Logistic Regression) on radiomics features from bpMRI (T2W, ADC, and LoG-filtered images)	AUC, ACC, Sensitivity, Specificity, Recall, F1-score, Feature selection results

Full List of Included Studies

[1] Shao, L.; Liu, Z.; Liu, J.; Yan, Y.; Sun, K.; Liu, X.; Lu, J.; Tian, J. Patient-level grading prediction of prostate cancer from mp-MRI via GMINet. *Comput. Biol. Med.* **2022**, 150, 106168.

[2] Bulten, W.; Balkenhol, M.; Belinga, J.-J. A.; Brilhante, A.; Cakir, A.; Egevad, L.; Eklund, M.; Farre, X.; Geronatisou, K.; Molinié, V.; et al. Artificial intelligence assistance significantly improves Gleason grading of prostate biopsies by pathologists. *Mod. Pathol.* **2021**, 34, 660–671.

[3] Pantanowitz, L.; Quiroga-Garza, G. M.; Bien, L.; Heled, R.; Laifenfeld, D.; Linhart, C.; Sandbank, J.; Shach, A. A.; Shalev, V.; Vecsler, M.; et al. An artificial intelligence algorithm for prostate cancer diagnosis in whole slide images of core needle biopsies: a blinded clinical validation and deployment study. *Lancet Digit. Health* **2020**, 2, e407–e416. Lancet Digit. Health 2020, 2, e407-e416.

[4] Jung, M.; Jin, M.-S.; Kim, C.; Lee, C.; Nikas, I. P.; Park, J. H.; Ryu, H. S. Artificial intelligence system shows performance at the level of uropathologists for the detection and grading of prostate cancer in core needle biopsy: an independent external validation study. *Mod. Pathol.* **2022**, 35, 1449–1457.

[5] Silva, S. P.; McIntire, P.; Alexander, M.; Gagnon, M.; Kevan, K.; Zhou, A. G.; Rizk, C.; Haines, K.; Siev, M.; Pirozzi, G.; et al. Independent external validation of a machine learning algorithm for prostate cancer detection and grading in whole slide images of core needle biopsies. Mod. Pathol. 2021, 34, 1134–1142.

[6] Perera, M.; Smith, L.; Thompson, I.; Breemer, G.; Papa, N.; Patel, M. I.; Swindle, P.; Smith, E. Advancing traditional prostate-specific antigen kinetics in the detection of prostate cancer: a machine learning model. *Eur. Urol.* **2021**, 79, 585–592.

[7] Ström, P.; Kartasalo, K.; Olsson, H.; Solorzano, L.; Delahunt, B.; Berney, D. M.; Bostwick, D.; Evans, A.; Grignon, D.; Humphrey, P. A.; et al. Artificial intelligence for diagnosis and grading of prostate cancer in biopsies: a population-based, diagnostic study. *Lancet Oncol.* **2020**, 21, 222–232.

[8] Bhattacharya, I.; Seetharaman, A.; Kunder, C.; Shao, W.; Chen, L. C.; Soerensen, S. J. C.; Wang, J. B.; Teslovich, N. C.; Fan, R. E.; Ghanouni, P.; et al. Selective identification and localization of indolent and aggressive prostate cancers via CorrSigNIA: an MRI-pathology correlation and deep learning

framework. *Med. Image Anal.* **2022**, 75, 102288.
[9] Marginean, F.; Arvidsson, I.; Simoulis, A.; Overgaard, N. C.; Aström, K.; Heyden, A.; Bjartell, A.; Krzyzanowska, A. An artificial intelligence-based support tool for automation and standardisation of Gleason grading in prostate biopsies. *Eur. Urol. Focus* **2021**, 7, 995–1001.
[10] Salman, M. E.; Cakirsoy Cakar, G.; Azimjonov, J.; Kosem, M.; Cedimoğlu, I. H. Automated prostate cancer grading and diagnosis system using deep learning-based Yolo object detection algorithm. *Expert Syst. Appl.* **2022**, 191, 116148.
[11] Winkel, D. J.; Tong, A.; Lou, B.; Kamen, A.; Comaniciu, D.; Disselhorst, J. A.; Rodríguez-Ruiz, A.; Huisman, H.; Szolar, D.; Shabunin, I.; et al. A novel deep learning based computer-aided diagnosis system improves the accuracy and efficiency of radiologists in reading biparametric magnetic resonance images of the prostate: results of a multireader, multicase study. *Invest. Radiol.* **2021**, 56, 605–613.
[12] Cani, A. K.; Hu, K.; Liu, C.-J.; Siddiqui, J.; Zheng, Y.; Han, S.; Nallandhighal, S.; Hovelson, D. H.; Xiao, L.; Pham, T.; et al. Development of a whole-urine, multiplexed, next-generation RNA-sequencing assay for early detection of aggressive prostate cancer. *Eur. Urol. Oncol.* **2022**, 5, 430–439.
[13] Hiremath, A.; Shiradkar, R.; Fu, P.; Mahran, A.; Rastinehad, A. R.; Tewari, A.; Tirumani, S. H.; Purysko, A.; Ponsky, L.; Madabhushi, A. An integrated nomogram combining deep learning, Prostate Imaging-Reporting and Data System (PI-RADS) scoring, and clinical variables for identification of clinically significant prostate cancer on biparametric MRI: a retrospective multicentre study. *Lancet Digit. Health* **2021**, 3, e445–e454.
[14] Peng, T.; Xiao, J.; Li, L.; Pu, B.; Niu, X.; Zeng, X.; Wang, Z.; Gao, C.; Li, C.; Chen, L.; et al. Can machine learning-based analysis of multiparameter MRI and clinical parameters improve the performance of clinically significant prostate cancer diagnosis? *Int. J. Comput. Assist. Radiol. Surg.* **2021**, 16, 2235–2249.
[15] Winkel, D. J.; Breit, H.-C.; Shi, B.; Boll, D. T.; Seifert, H.-H.; Wetterauer, C. Predicting clinically significant prostate cancer from quantitative image features including compressed sensing radial MRI of prostate perfusion using machine learning: comparison with PI-RADS v2 assessment scores. *Quant. Imaging Med. Surg.* **2020**, 10, 808–823.
[16] Pinckaers, H.; Bulten, W.; van der Laak, J.; Litjens, G. Detection of prostate cancer in whole-slide images through end-to-end training with image-level labels. *IEEE Trans. Med. Imaging* **2021**, 40, 1818–1827.
[17] Yang, B.; Xiao, Z. A multi-channel and multi-spatial attention convolutional neural network for prostate cancer ISUP grading. *Appl. Sci.* **2021**, 11, 4321.
[18] Connell, S. P.; Mills, R.; Pandha, H.; Morgan, R.; Cooper, C. S.; Clark, J.; Brewer, D. S.; et al. Integration of urinary EN2 protein & cell-free RNA data in the development of a multivariable risk model for the detection of prostate cancer prior to biopsy. *Cancers* **2021**, 13, 2102.
[19] Gaudiano, C.; Mottola, M.; Bianchi, L.; Corcioni, B.; Cattabriga, A.; Cocozza, M. A.; Palmeri, A.; Coppola, F.; Giunchi, F.; Schiavina, R.; et al. Beyond multiparametric MRI and towards radiomics to detect prostate cancer: a machine learning model to predict clinically significant lesions. *Cancers* **2022**, 14, 6156.
[20] Chen, S.; Zhang, H.; Yang, X.; Chen, N.; Chen, Z.; Liu, S. Noninvasive prostate cancer diagnosis based on urine Raman spectroscopy and convolutional neural network. *Adv. Intell. Syst.* **2021**, 3, 2000090.
[21] Dai, Y.; Wang, Y.; Cao, Y.; Yu, P.; Zhang, L.; Liu, Z.; Ping, Y.; Wang, D.; Zhang, G.; Sang, Y.; et al. A multivariate diagnostic model based on urinary EpCAM-CD9-positive extracellular vesicles for prostate cancer diagnosis. *Front. Oncol.* **2021**, 11, 777684.
[22] Gentile, F.; Ferro, M.; Della Ventura, B.; La Civita, E.; Liotti, A.; Cennamo, M.; Bruzzese, D.; Velotta, R.; Terracciano, D. Optimized identification of high-grade prostate cancer by combining different PSA molecular forms and PSA density in a deep learning model. *Diagnostics* **2021**, 11, 335.
[23] Liu, Y.; Zheng, H.; Liang, Z.; Miao, Q.; Brisbane, W. G.; Marks, L. S.; Raman, S. S.; Reiter, R. E.; Yang,

G.; Sung, K. Textured-based deep learning in prostate cancer classification with 3T multiparametric MRI: comparison with PI-RADS-based classification. *Diagnostics* **2021**, 11, 1785.
[24] Gravina, M.; Spirito, L.; Celentano, G.; Capece, M.; Creta, M.; Califano, G.; Colla Ruvolo, C.; Morra, S.; Imbriaco, M.; Di Bello, F.; et al. Machine learning and clinical-radiological characteristics for the classification of prostate cancer in PI-RADS 3 lesions. *Diagnostics* **2022**, 12, 1565.
[25] Grosset, A.-A.; Dallaire, F.; Nguyen, T.; Birlea, M.; Wong, J.; Daoust, F.; Roy, N.; Kougioumoutzakis, A.; Azzi, F.; Aubertin, K.; et al. Identification of intraductal carcinoma of the prostate on tissue specimens using Raman micro-spectroscopy: a diagnostic accuracy case-control study with multicolor validation. *PLoS Med.* **2020**, 17, e1003281.
[26] Kim, H.; Park, S.; Jeong, I. G.; Song, S. H.; Jeong, Y.; Kim, C.-S.; Lee, K. H. Noninvasive precision screening of prostate cancer by urinary multimarker sensor and artificial intelligence analysis. *ACS Nano* **2021**, 15, 4054–4065.
[27] Qiu, Y.; Hu, Y.; Kong, P.; Xie, H.; Zhang, X.; Cao, J.; Wang, T.; Lei, B. Automatic prostate Gleason grading using pyramid semantic parsing network in digital histopathology. *Front. Oncol.* **2022**, 12, 772403.
[28] Deng, X.; Li, T.; Mo, L.; Wang, F.; Ji, J.; He, X.; Mohamud, B. H.; Pradhan, S.; Cheng, J. Machine learning model for the prediction of prostate cancer in patients with low prostate-specific antigen levels: a multicenter retrospective analysis. *Front. Oncol.* **2022**, 12, 985940.
[29] Huang, W.; Randhawa, R.; Jain, P.; Iczkowski, K. A.; Hu, R.; Hubbard, S.; Eickhoff, J.; Basu, H.; Roy, R. Development and validation of an artificial intelligence-powered platform for prostate cancer grading and quantification. *JAMA Netw. Open* **2021**, 4, e2132554.
[30] Nagpal, K.; Foote, D.; Tan, F.; Liu, Y.; Chen, P.-H. C.; Steiner, D. F.; Manoj, N.; Olson, N.; Smith, J. L.; Mohtashamian, A.; et al. Development and validation of a deep learning algorithm for Gleason grading of prostate cancer from biopsy specimens. *JAMA Oncol.* **2020**, 6, 1372–1380.
[31] Aldoj, N.; Lukas, S.; Dewey, M.; Penzkofer, T. Semi-automatic classification of prostate cancer on multi-parametric MR imaging using a multi-channel 3D convolutional neural network. *Eur. Radiol.* **2019**, 30, 1243–1253.
[32] Khosravi, P.; Lysandrou, M.; Eljalby, M.; Li, Q.; Kazemi, E.; Zisimopoulos, P.; Sigaras, A.; Brendel, M.; Barnes, J.; Ricketts, C.; et al. A deep learning approach to diagnostic classification of prostate cancer using pathology–radiology fusion. *J. Magn. Reson. Imaging* **2021**, 54, 462–471.
[33] Song, S. H.; Kim, H.; Kim, J. K.; Lee, H.; Oh, J. J.; Lee, S.-C.; Jeong, S. J.; Hong, S. K.; Lee, J.; Yoo, S.; et al. A smart, practical, deep learning-based clinical decision support tool for patients in the prostate-specific antigen gray zone: model development and validation. *J. Am. Med. Inform. Assoc.* **2022**, 29, 1949–1957.
[34] Duran-Lopez, L.; Dominguez-Morales, J. P.; Conde-Martin, A. F.; Vicente-Diaz, S.; Linares-Barranco, A. PROMETEO: a CNN-based computer-aided diagnosis system for WSI prostate cancer detection. *IEEE Access* **2020**, 8, 186613–186629.
[35] Yi, Z.; Hu, S.; Lin, X.; Zou, Q.; Zou, M.; Zhang, Z.; Xu, L.; Jiang, N.; Zhang, Y. Machine learning-based radiomics models for prediction of invisible intraprostatic lesions on 68Ga-PSMA-11 PET in patients with primary prostate cancer. *Eur. J. Nucl. Med. Mol. Imaging* **2021**, 49, 1523–1532.
[36] Pellicer-Valero, O. J.; Marenco Jiménez, J. L.; Gonzalez-Perez, V.; Casanova Ramón-Borja, J. L.; Martín García, I.; Barrios Benito, M.; Pelechano Gómez, P.; Rubio-Briones, J.; Ruperez, M. J.; Martín-Guerrero, J. D. Deep learning for fully automatic detection, segmentation, and Gleason grade estimation of prostate cancer in multiparametric magnetic resonance images. *Sci. Rep.* **2022**, 12, 2975.
[37] Arif, M.; Schoots, I. G.; Castillo Tovar, J.; Bangma, C. H.; Krestin, G. P.; Roobol, M. J.; Niessen, W.; Veenland, J. F. Clinically significant prostate cancer detection and segmentation in low-risk patients using a convolutional neural network on multi-parametric MRI. *Eur. Radiol.* **2020**, 30, 6582–6592.
[38] Bleker, J.; Kwee, T. C.; Rouw, D.; Roest, C.; Borstlap, J.; de Jong, I. J.; Dierckx, R. A. J. O.; Huisman, H.; Yakar, D. A deep learning masked segmentation alternative to manual segmentation in biparametric

MRI prostate cancer radiomics. *Eur. Radiol.* **2022**, 32, 6526–6535.
[39] Johnson, H.; Guo, J.; Zhang, X.; Zhang, H.; Simoulis, A.; Wu, A. H. B.; Xia, T.; Li, F.; Tan, W.; Johnson, A.; et al. Development and validation of a 25-Gene Panel urine test for prostate cancer diagnosis and potential treatment follow-up. *BMC Med.* **2020**, 18, 376.
[40] Bulten, W.; Kartasalo, K.; Chen, P.-H. C.; Ström, P.; Pinckaers, H.; Nagpal, K.; Cai, Y.; Steiner, D. F.; van Boven, H.; Vink, R.; et al. Artificial intelligence for diagnosis and Gleason grading of prostate cancer: the PANDA challenge. *Nat. Med.* **2022**, 28, 154–163.
[41] da Silva, L. M. S.; Pereira, E. M.; Salles, P. G. O.; Godrich, R.; Cabañas, R.; Sávio, J.; Ferreira, A.; Faria, B.; Kunz, J.; Rothrock, B.; et al. Independent real-world application of a clinical-grade automated prostate cancer detection system. *J. Pathol.* **2021**, 254, 147–158.
[42] Singhal, N.; Soni, S.; Bonthu, S.; Chattopadhyay, N.; Samanta, P.; Joshi, U.; Joiera, A.; Chharchhodawala, T.; Agarwal, A.; Desai, M.; et al. A deep learning system for prostate cancer diagnosis and grading in whole slide images of core needle biopsies. *Sci. Rep.* **2022**, 12, 3383.
[43] Steiner, D. F.; Nagpal, K.; Sayres, R.; Foote, D. J.; Wedin, B. D.; Pearce, A.; Cai, C. J.; Winter, S. R.; Symonds, M.; Yatziv, L.; et al. Evaluation of the use of combined artificial intelligence and pathologist assessment to review and grade prostate biopsies. *JAMA Netw. Open* **2020**, 3, e2023267.
[44] Marron-Esquivel, J. M.; Duran-Lopez, L.; Linares-Barranco, A.; Dominguez-Morales, J. P. A comparative study of the inter-observer variability on Gleason grading against Deep Learning-based approaches for prostate cancer. *Comput. Biol. Med.* **2023**, 154, 106528.
[45] Xiang, J.; Wang, X.; Wang, X.; Zhang, J.; Yang, S.; Yang, W.; Han, X.; Liu, Y. Automatic diagnosis and grading of Prostate Cancer with weakly supervised learning on whole slide images. *Comput. Biol. Med.* **2023**, 152, 106340.
[46] Wang, Y.; Qian, H.; Shao, X.; Zhang, H.; Liu, S.; Pan, J.; Xue, W. Multimodal convolutional neural networks based on the Raman spectra of serum and clinical features for the early diagnosis of prostate cancer. *Comput. Biol. Med.* **2023**, 156, 106723.
[47] Liu, F.; Zhao, Y.; Song, J.; Tu, G.; Liu, Y.; Peng, Y.; Mao, J.; Yan, C.; Wang, R. A hybrid classification model with radiomics and CNN for high and low grading of prostate cancer Gleason score on mp-MRI. *Displays* **2024**, 83, 102703.
[48] Bashkanov, O.; Rak, M.; Meyer, A.; Engelage, L.; Lumiani, A.; Muschter, R.; Hansen, C. Automatic detection of prostate cancer grades and chronic prostatitis in biparametric MRI. *Comput. Methods Programs Biomed.* **2023**, 239, 107624.
[49] Ramirez-Mena, A.; Andres-Leon, E.; Alvarez-Cubero, M. J.; Anguita-Ruiz, A.; Martinez-Gonzalez, L. J.; Alcala-Fdez, J. Explainable artificial intelligence to predict and identify prostate cancer tissue by gene expression. *Comput. Methods Programs Biomed.* **2023**, 240, 107719.
[50] Harder, C.; Pryalukhin, A.; Quaas, A.; Eich, M.-L.; Tretiakova, M.; Klein, S.; Seper, A.; Heidenreich, A.; Netto, G. J.; Hulla, W.; et al. Enhancing prostate cancer diagnosis: artificial intelligence–driven virtual biopsy for optimal magnetic resonance imaging-targeted biopsy approach and Gleason grading strategy. *Mod. Pathol.* **2024**, 37, 100564.
[51] Jaouen, T.; Souchon, R.; Moldovan, P. C.; Bratan, F.; Duran, A.; Hoang-Dinh, A.; Di Franco, F.; Debeer, S.; Dubreuil-Chambardel, M.; Arfi, N.; et al. Characterization of high-grade prostate cancer at multiparametric MRI using a radiomic-based computer-aided diagnosis system as standalone and second reader. *Diagn. Interv. Imaging* **2023**, 104, 465–476.
[52] Saha, A.; Bosma, J. S.; Twilt, J. J.; van Ginneken, B.; Bjartell, A.; Padhani, A. R.; Bonekamp, D.; Villeirs, G.; Salomon, G.; Giannarini, G.; et al. Artificial intelligence and radiologists in prostate cancer detection on MRI (PI-CAI): an international, paired, noninferiority, confirmatory study. *Lancet Oncol.* **2024**, 25, 879–887.
[53] Li, S.-T.; Zhang, L.; Guo, P.; Pan, H.-Y.; Chen, P.-Z.; Xie, H.-F.; Xie, B.-K.; Chen, J.; Lai, Q.-Q.; Li, Y.-Z.; et al. Prostate cancer of magnetic resonance imaging automatic segmentation and detection of based on 3D-Mask RCNN. *Biomed. Signal Process. Control* **2024**, 90, 105853.

[54] Sowmya, D.; Bhavani, S. A.; Sasank, V. V. S.; Rao, T. S. Prostate cancer classification using adaptive swarm intelligence based deep attention neural network. *Biomed. Signal Process. Control* **2024**, 92, 106060.
[55] Kong, F.; Wang, X.; Xiang, J.; Yang, S.; Wang, X.; Yue, M.; Zhang, J.; Zhao, J.; Han, X.; Dong, Y.; et al. Federated attention consistent learning models for prostate cancer diagnosis and Gleason grading. *Comput. Struct. Biotechnol. J.* **2024**, 23, 1439–1449.
[56] Chandrasekhara, S. P. R.; Kabadi, M. G.; Srivnay. Wearable IoT based diagnosis of prostate cancer using GLCM-multiclass SVM and SIFT-multiclass SVM feature extraction strategies. *Int. J. Pervasive Comput. Commun.* **2024**, 20, 19–36.
[57] van den Kroonenberg, D. L.; Jager, A.; Garrido-Utrilla, A.; Reitsma, J. B.; Postema, A. W.; Beerlage, H. P.; Oddens, J. R. Clinical validation of multiparametric ultrasound for detecting clinically significant prostate cancer using computer-aided diagnosis: a direct comparison with the magnetic resonance imaging pathway. *Eur. Urol. Open Sci.* **2024**, 66, 1–7.
[58] Zaridis, D. I.; Mylona, E.; Tsiknakis, N.; Tachos, N. S.; Matsopoulos, G. K.; Marias, K.; Tsiknakis, M.; Fotiadis, D. I. ProLesA-Net: A multi-channel 3D architecture for prostate MRI lesion segmentation with multi-scale channel and spatial attentions. *Patterns* **2024**, 5, 100992.
[59] Sanjid, K. S.; Junayed, M. S. S.; Hossain, M. T.; Wang, Y.-L.; Uddin, M. M.; Haque, S. A. From pixels to pathology: A novel dual-pathway multi-scale hierarchical upsampling network for MRI-based prostate zonal segmentation. *Intell. Syst. Appl.* **2024**, 22, 200382.
[60] Chen, Z.-L.; Huang, Z.-C.; Lin, S.-S.; Li, Z.-H.; Dou, R.-L.; Xu, Y.; Jiang, S.-Q.; Li, M.-Q. Clinical value of a radiomics model based on machine learning for the prediction of prostate cancer. *J. Int. Med. Res.* **2024**, 52, 03000605241275338.
[61] Kondejkar, T.; Al-Heejawi, S. M. A.; Breggia, A.; Ahmad, B.; Christman, R.; Ryan, S. T.; Amal, S. Multi-scale digital pathology patch-level prostate cancer grading using deep learning: use case evaluation of DiagSet dataset. *Bioengineering* **2024**, 11, 624.
[62] Ao, J.; Shao, X.; Liu, Z.; Liu, Q.; Xia, J.; Shi, Y.; Qi, L.; Pan, J.; Ji, M. Stimulated Raman scattering microscopy enables Gleason scoring of prostate core needle biopsy by a convolutional neural network. *Cancer Res.* **2023**, 83, 641–651.
[63] Zhang, J.; Kang, F.; Gao, J.; Jiao, J.; Quan, Z.; Ma, S.; Li, Y.; Guo, S.; Li, Z.; Jing, Y.; et al. A prostate-specific membrane antigen PET-based approach for improved diagnosis of prostate cancer in Gleason grade group 1: a multicenter retrospective study. *J. Nucl. Med.* **2023**, 64, 1750–1757.
[64] Alici-Karaca, D.; Akay, B. An efficient deep learning model for prostate cancer diagnosis. *IEEE Access* **2024**, 12, 151234–151248.
[65] Song, Z.; Zhang, W.; Jiang, Q.; Deng, L.; Du, L.; Mou, W.; Lai, Y.; Zhang, W.; Yang, Y.; Lim, J.; et al. Artificial intelligence-aided detection for prostate cancer with multimodal routine health check-up data: an Asian multi-center study. *Int. J. Surg.* **2023**, 109, 3849–3861.
[66] Gaudiano, C.; Mottola, M.; Bianchi, L.; Corcioni, B.; Braccischi, L.; Giunchi, F.; Schiavina, R.; Fanti, S.; Fiorentino, M.; Brunocilla, E.; et al. Radiomics-Based Prediction of Prostate Cancer in PI-RADS 3 Lesions: A Proof-of-Concept Study. *Cancers* **2023**, 15, 4796.
[67] El-Melegy, M.; Mamdouh, A.; Ali, S.; Badawy, M.; El-Ghar, M. A.; Alghamdi, N. S.; El-Baz, A. Prostate Cancer Diagnosis via Visual Representation of Tabular Data and Deep Transfer Learning. *Bioengineering* **2024**, 11, 635.
[68] Yang, E.; Shankar, K.; Kumar, S.; Seo, C.; Moon, I. Equilibrium Optimization Algorithm with Deep Learning-Based Prostate Cancer Detection and Classification Model on Magnetic Resonance Images. *Diagnostics* **2023**, 13, 3584.
[69] Paproski, R. J.; Pink, D.; Sosnowski, D. L.; Vasquez, C.; Lewis, J. D. Extracellular vesicle machine learning platform (EVMAP) to predict high-grade prostate cancer: a validation study in a prospective cohort. *Mol. Oncol.* **2023**, 17, 613–628.
[70] Cai, J. C.; Nakai, H.; Kuanar, S.; Froemming, A. T.; Bolan, C. W.; Kawashima, A.; Takahashi, H.; Mynderse, L. A.; Dora, C. D.; Humphreys, M. R.; et al. Fully Automated Deep Learning Model to Detect

Clinically Significant Prostate Cancer at MRI. *Radiology* **2024**, 312, e232635.
[71] Qi, X.; Wang, K.; Feng, B.; Sun, X.; Yang, J.; Hu, Z.; Zhang, M.; Lv, C.; Jin, L.; Zhou, L.; et al. Comparison of machine learning models based on multi-parametric magnetic resonance imaging and ultrasound videos for the prediction of prostate cancer. *Front. Oncol.* **2023**, 13, 1157949.
[72] Liu, J.-C.; Ruan, X.-H.; Chun, T.-T.; Yao, C.; Huang, D.; Wong, H.-L.; Lai, C.-T.; Tsang, C.-F.; Ho, S.-H.; Ng, T.-L.; et al. MRI T2w Radiomics-Based Machine Learning Models in Imaging Simulated Biopsy Add Diagnostic Value to PI-RADS in Predicting Prostate Cancer: A Retrospective Diagnostic Study. *Cancers* **2024**, 16, 2944.
[73] Gavade, A. B.; Nerli, R. B.; Pol, S. S.; Kumar, N.; Gavade, P. A.; Rizvi, S. T. H. Automated Detection and Classification of Prostate Cancer from Multiparametric MRI Using Deep Learning Techniques. Diagnostics 2023, 13, 2527.
[74] Horasan, A.; Gunes, A. Advancing Prostate Cancer Diagnosis: A Deep Learning Approach for Enhanced Detection in MRI Images. *Diagnostics* **2024**, 14, 1871.
[75] Zhang, M.; Liu, Y.; Yao, J.; Wang, K.; Tu, J.; Hu, Z.; Jin, Y.; Du, Y.; Sun, X.; Chen, L.; et al. Value of machine learning-based transrectal multimodal ultrasound combined with PSA-related indicators in the diagnosis of clinically significant prostate cancer. *Front. Endocrinol.* **2023**, 14, 1137322.
[76] Li, S.; Zheng, T.; Fan, Z.; Qu, H.; Wang, J.; Bi, J.; Lv, Q.; Zhang, G.; Cui, X.; Zhao, Y. A dynamic-static combination model based on radiomics features for prostate cancer using multiparametric MRI. *Phys. Med. Biol.* **2023**, 68, 015008.
[77] Mehmood, M.; Abbasi, S. H.; Aurangzeb, K.; Majeed, M. F.; Anwar, M. S.; Alhussein, M. A classifier model for prostate cancer diagnosis using CNNs and transfer learning with multi-parametric MRI. *Front. Oncol.* **2023**, 13, 1225490.
[78] Zhao, Y.-Y.; Xiong, M.-L.; Liu, Y.-F.; Duan, L.-J.; Chen, J.-L.; Xing, Z.; Lin, Y.-S.; Chen, T.-H. Magnetic resonance imaging radiomics-based prediction of clinically significant prostate cancer in equivocal PI-RADS 3 lesions in the transitional zone. *Front. Oncol.* **2023**, 13, 1247682.
[79] Hamm, C. A.; Baumgärtner, G. L.; Biessmann, F.; Beetz, N. L.; Hartenstein, A.; Savic, L. J.; Froböse, K.; Dräger, F.; Schallenberg, S.; Rudolph, M.; et al. Interactive Explainable Deep Learning Model Informs Prostate Cancer Diagnosis at MRI. *Radiology* **2023**, 307, e222276.
[80] Huang, X.; Wang, Q.; He, J.; Ban, C.; Zheng, H.; Chen, H.; Zhu, X. Fast Multiphoton Microscopic Imaging Joint Image Super-Resolution for Automated Gleason Grading of Prostate Cancers. *J. Biophotonics* **2024**, 17, e202400233.
[81] Li, Y.; Wynne, J.; Wang, J.; Roper, J.; Chang, C.-W.; Patel, A. B.; Shelton, J.; Liu, T.; Mao, H.; Yang, X. MRI-based prostate cancer classification using 3D efficient capsule network. *Med. Phys.* **2024**, 51, 4748–4758.
[82] Li, H.; Liu, H.; von Busch, H.; Grimm, R.; Huisman, H.; Tong, A.; Winkel, D.; Penzkofer, T.; Shabunin, I.; Choi, M. H.; et al. Deep Learning–based Unsupervised Domain Adaptation via a Unified Model for Prostate Lesion Detection Using Multisite Biparametric MRI Datasets. *Radiol. Artif. Intell.* **2024**, 6, e230521.
[83] Jiang, K.-W.; Song, Y.; Hou, Y.; Zhi, R.; Zhang, J.; Bao, M.-L.; Li, H.; Yan, X.; Xi, W.; Zhang, C.-X.; et al. Performance of Artificial Intelligence-Aided Diagnosis System for Clinically Significant Prostate Cancer with MRI: A Diagnostic Comparison Study. *J. Magn. Reson. Imaging* **2023**, 57, 1352–1364.
[84] Liu, Y.; Fan, Y.; Wang, X.; Huang, T.; Zhang, C.; Chen, R.; Zhang, M.; Li, M.; Wang, W.; Liang, C. Development and Validation of Interpretable Machine Learning Models for Clinically Significant Prostate Cancer Diagnosis in Patients With Lesions of PI-RADS v2.1 Score ≥3. *J. Magn. Reson. Imaging* **2024**, 60, 2134–2143.
[85] Mannas, M. P.; Deng, F. M.; Ion-Margineanu, A.; Morales, B.; Jones, D.; Taouli, B.; Lewis, S.; Haines, G. K., III; Fenyő, D.; Manova-Todorova, K.; et al. Stimulated Raman Histology Interpretation by Artificial Intelligence Provides Near-Real-Time Pathologic Feedback for Unprocessed Prostate Biopsies. *J. Urol.* **2024**, 211, 384–391.

[86] Zhao, L.; Bao, J.; Qiao, X.; Jin, P.; Ji, Y.; Li, Z.; Zhang, J.; Su, Y.; Ji, L.; Shen, J.; et al. Predicting clinically significant prostate cancer with a deep learning approach: a multicentre retrospective study. *Eur. J. Nucl. Med. Mol. Imaging* **2023**, 50, 727–741.
[87] Roest, C.; Yakar, D.; Sitar, D. I. R.; Bosma, J. S.; Row, D. B.; Fransen, S. J.; Huisman, H.; Kwee, T. C. Multimodal AI Combining Clinical and Imaging Inputs Improves Prostate Cancer Detection. *Invest. Radiol.* **2024**, 59, 854–860.
[88] Fei, X.; Du, X.; Wang, J.; Liu, J.; Gong, Y.; Zhao, Z.; Cao, Z.; Fu, Q.; Zhu, Y.; Dong, L.; et al. Precise diagnosis and risk stratification of prostate cancer by comprehensive serum metabolic fingerprints: a prediction model study. *Int. J. Surg.* **2024**, 110, 1454–1465.
[89] Sun, Z.; Wang, K.; Wu, C.; Chen, Y.; Kong, Z.; She, L.; Song, B.; Luo, N.; Wu, P.; Wang, X.; et al. Using an artificial intelligence model to detect and localize visible clinically significant prostate cancer in prostate magnetic resonance imaging: a multicenter external validation study. *Quant. Imaging Med. Surg.* **2024**, 14, 43–60.
[90] Li, M.; Ding, N.; Yin, S.; Lu, Y.; Ji, Y.; Jin, L. Enhancing automatic prediction of clinically significant prostate cancer with deep transfer learning 2.5-dimensional segmentation on bi-parametric magnetic resonance imaging (bp-MRI). *Quant. Imaging Med. Surg.* **2024**, 14, 4893–4902.
[91] Schrader, A.; Netzer, N.; Hielscher, T.; Görtz, M.; Zhang, K. S.; Schütz, V.; Stenzinger, A.; Hohenfellner, M.; Schlemmer, H.-P.; Bonekamp, D. Prostate cancer risk assessment and avoidance of prostate biopsies using fully automatic deep learning in prostate MRI: comparison to PI-RADS and integration with clinical data in nomograms. *Eur. Radiol.* **2024**, 34, 7414–7425.
[92] Jafari, E.; Zarei, A.; Dadgar, H.; Keshavarz, A.; Manafi-Farid, R.; Rostami, H.; Assadi, M. A convolutional neural network-based system for fully automatic segmentation of whole-body [68Ga]Ga-PSMA PET images in prostate cancer. *Eur. J. Nucl. Med. Mol. Imaging* **2024**, 51, 1177–1188.
[93] Yang, F.; Wang, C.; Shen, J.; Ren, Y.; Yu, F.; Luo, W.; Su, X., End-to-end ^{18}F PSMA-1007 PET/CT radiomics-based pipeline for predicting ISUP grade group in prostate cancer. *Abdominal Radiology* **2025,** *50* (4), 1641-1652.
[94] Marvaso, G.; Isaksson, L. J.; Zaffaroni, M.; Vincini, M. G.; Summers, P. E.; Pepa, M.; Corrao, G.; Mazzola, G. C.; Rotondi, M.; Mastroleo, F.; et al. Can we predict pathology without surgery? Weighing the added value of multiparametric MRI and whole prostate radiomics in integrative machine learning models. *Eur. Radiol.* **2024**, 34, 6241–6253.
[95] Liu, G.; Pan, S.; Zhao, R.; Zhou, H.; Chen, J.; Zhou, X.; Xu, J.; Zhou, Y.; Xue, W.; Wu, G. The added value of AI-based computer-aided diagnosis in classification of cancer at prostate MRI. *Eur. Radiol.* **2023**, 33, 5118–5130.
[96] Gao, Y.; Vali, M., Combination of Deep and Statistical Features of the Tissue of Pathology Images to Classify and Diagnose the Degree of Malignancy of Prostate Cancer. *Journal of Imaging Informatics in Medicine* **2025, 38(4), 2241-2259**.
[97] Wang, X.; Xie, Y.; Zheng, X.; Liu, B.; Chen, H.; Li, J.; Ma, X.; Xiang, J.; Weng, G.; Zhu, W.; et al. A prospective multi-center randomized comparative trial evaluating outcomes of transrectal ultrasound (TRUS)-guided 12-core systematic biopsy, mpMRI-targeted 12-core biopsy, and artificial intelligence ultrasound of prostate (AIUSP) 6-core targeted biopsy for prostate cancer diagnosis. *World J. Urol.* **2023**, 41, 653–662.
[98] Huang, J.; He, C.; Xu, P.; Song, B.; Zhao, H.; Yin, B.; He, M.; Lu, X.; Wu, J.; Wang, H. Development and validation of a clinical-radiomics model for prediction of prostate cancer: a multicenter study. *World J. Urol.* **2024**, 42, 275.
[99] Yang, Z.; Wang, X.; Xiang, J.; Zhang, J.; Yang, S.; Wang, X.; Yang, W.; Li, Z.; Han, X.; Liu, Y. The devil is in the details: a small-lesion sensitive weakly supervised learning framework for prostate cancer detection and grading. *Virchows Arch.* **2023**, 482, 525–538.
[100] Balaha, H. M.; Shaban, A. O.; El-Gendy, E. M.; Saafan, M. M. Prostate cancer grading framework based on deep transfer learning and Aquila optimizer. *Neural Comput. Appl.* **2024**, 36, 7877–7902.

[101] Zhang, H.; Ji, J.; Liu, Z.; Lu, H.; Qian, C.; Wei, C.; Chen, S.; Lu, W.; Wang, C.; Xu, H.; et al. Artificial intelligence for the diagnosis of clinically significant prostate cancer based on multimodal data: a multicenter study. *BMC Med.* **2023**, 21, 270.
[102] Wang, Y.; Liu, W.; Chen, Z.; Zang, Y.; Xu, L.; Dai, Z.; Zhou, Y.; Zhu, J. A noninvasive method for predicting clinically significant prostate cancer using magnetic resonance imaging combined with PRKY promoter methylation level: a machine learning study. *BMC Med. Imaging* **2024**, 24, 60.
[103] Zhao, W.; Hou, M.; Wang, J.; Song, D.; Niu, Y. Interpretable machine learning model for predicting clinically significant prostate cancer: integrating intratumoral and peritumoral radiomics with clinical and metabolic features. *BMC Med. Imaging* **2024**, 24, 353.
[104] Chen, G.; Dai, X.; Zhang, M.; Tian, Z.; Jin, X.; Mei, K.; Huang, H.; Wu, Z. Machine learning-based prediction model and visual interpretation for prostate cancer. *BMC Urol.* **2023**, 23, 164.
[105] Talaat, F. M.; El-Sappagh, S.; Alnowaiser, K.; Hassan, E. Improved prostate cancer diagnosis using a modified ResNet50-based deep learning architecture. *BMC Med. Inform. Decis. Mak.* **2024**, 24, 23.
[106] Zheng, H.; Hung, A. L. Y.; Miao, Q.; Song, W.; Scalzo, F.; Raman, S. S.; Zhao, K.; Sung, K. AtPCa-Net: anatomical-aware prostate cancer detection network on multi-parametric MRI. *Sci. Rep.* **2024**, 14, 5740.
[107] Sun, Z.; Wang, K.; Kong, Z.; Xing, Z.; Chen, Y.; Luo, N.; Yu, Y.; Song, B.; Wu, P.; Wang, X.; et al. A multicenter study of artificial intelligence-aided software for detecting visible clinically significant prostate cancer on mpMRI. *Insights Imaging* **2023**, 14, 72.
[108] Jin, L.; Yu, Z.; Gao, F.; Li, M. T2-weighted imaging-based deep-learning method for noninvasive prostate cancer detection and Gleason grade prediction: a multicenter study. *Insights Imaging* **2024**, 15, 106.
[109] Yu, R.; Jiang, K.; Bao, J.; Hou, Y.; Yi, Y.; Wu, D.; Song, Y.; Hu, C.-H.; Yang, G.; Zhang, Y.-D. PI-RADSAI: introducing a new human-in-the-loop AI model for prostate cancer diagnosis based on MRI. *Br. J. Cancer* **2023**, 128, 1019–1029.
[110] Bao, J.; Hou, Y.; Qin, L.; Zhi, R.; Wang, X.-M.; Shi, H.-B.; Sun, H.-Z.; Hu, C.-H.; Zhang, Y.-D. High-throughput precision MRI assessment with integrated stack-ensemble deep learning can enhance the preoperative prediction of prostate cancer Gleason grade. *Br. J. Cancer* **2023**, 128, 1267–1277.
[111] Shi, F.; Qi, Y.; Jiang, S.; Sun, N.; Deng, C. Hollow Core-Shell Metal Oxide Heterojunctions for the Urinary Metabolic Fingerprint-Based Noninvasive Diagnostic Strategy. *Anal. Chem.* **2023**, 95, 7312–7319.
[112] Tolkach, Y.; Ovtcharov, V.; Pryalukhin, A.; Eich, M.-L.; Gaisa, N. T.; Braun, M.; Radzhabov, A.; Quaas, A.; Hammerer, P.; Dellmann, A.; et al. An international multi-institutional validation study of the algorithm for prostate cancer detection and Gleason grading. *npj Precis. Oncol.* **2023**, 7, 77.
[113] Huo, X.; Ong, K. H.; Lau, K. W.; Gole, L.; Young, D. M.; Tan, C. L.; Zhu, X.; Zhang, C.; Zhang, Y.; Li, L.; et al. A comprehensive AI model development framework for consistent Gleason grading. *Commun. Med.* **2024**, 4, 85.
[114] Alzate-Grisales, J. A.; Mora-Rubio, A.; García-García, F.; Tabares-Soto, R.; de la Iglesia-Vayá, M. SAM-UNETR: Clinically Significant Prostate Cancer Segmentation Using Transfer Learning From Large Model. *IEEE Access* **2023**, 11, 118491–118502.
[115] Pan, Z.; Wang, Y.; Du, X.; Zhou, Y.; Dong, B.; Pan, J.; Wang, X.; Huang, C.; Yang, S.; Xu, X.; et al. Ferric Nanoparticle-Assisted Laser Desorption/Ionization Mass Spectrometry for Prostate Fluid Metabolic Fingerprint Combined with Machine Learning for Precise Diagnosis of Prostate Cancer with High Gleason Score. *Small Methods* **2024**, *8*, 2301684.
[116] Martelin, N.; De Witt, B.; Chen, B.; Eschwege, P. Development and validation of an imageless machine-learning algorithm for the initial screening of prostate cancer. *Prostate* **2024**, 84, 842–849.
[117] Jia, X.; Salmon, R.; Mulliqi, N.; Khan, U.; Wang, Y.; Blilie, A.; Olsson, H.; Pedersen, B. G.; Sørensen, K. D.; Ulhøi, B. P.; et al. Physical Color Calibration of Digital Pathology Scanners for Robust Artificial Intelligence–Assisted Cancer Diagnosis. *Mod. Pathol.* **2025**, 38, 100715.
[118] Smelik, M.; Diaz-Roncero Gonzalez, D.; An, X.; Heer, R.; Henningsohn, L.; Li, X.; Wang, H.; Zhao,

Y.; Benson, M. Combining Spatial Transcriptomics, Pseudotime, and Machine Learning Enables Discovery of Biomarkers for Prostate Cancer. *Cancer Res.* **2025**, 85, 2241–2253.
[119] Du, X.; Hao, S.; Olsson, H.; Kartasalo, K.; Mulliqi, N.; Rai, B.; Menges, D.; Heintz, E.; Egevad, L.; Eklund, M.; et al. Effectiveness and Cost-effectiveness of Artificial Intelligence-assisted Pathology for Prostate Cancer Diagnosis in Sweden: A Microsimulation Study. *Eur. Urol. Oncol.* **2025**, 8, 80–86.
[120] Twilt, J. J.; Saha, A.; Bosma, J. S.; Padhani, A. R.; Bonekamp, D.; Giannarini, G.; van den Bergh, R.; Kasivisvanathan, V.; Obuchowski, N.; Yakar, D.; et al. AI-Assisted vs Unassisted Identification of Prostate Cancer in Magnetic Resonance Images. *JAMA Netw. Open* **2025**, 8, e2515672.
[121] Choi, J. Y.; Park, S.; Shim, J. S.; Park, H. J.; Kuh, S. U.; Jeong, Y.; Park, M. G.; Noh, T. I.; Yoon, S. G.; Park, Y. M.; et al. Explainable artificial intelligence-driven prostate cancer screening using exosomal multi-marker based dual-gate FET biosensor. *Biosens. Bioelectron.* **2025**, 267, 116773.
[122] Sun, Z.; Wang, K.; Gao, G.; Wang, H.; Wu, P.; Li, J.; Zhang, X.; Wang, X. Assessing the Performance of Artificial Intelligence Assistance for Prostate MRI: A Two-Center Study Involving Radiologists With Different Experience Levels. *J. Magn. Reson. Imaging* **2025**, 61, 2234–2245.
[123] Padhani, A. R.; Papanikolaou, N. AI and human interactions in prostate cancer diagnosis using MRI. *Eur. Radiol.* **2025**, 35, 5695–5700.
[124] Zhong, X.; Yang, Y.; He, H.; Xiong, Y.; Zhong, M.; Wang, S.; Xia, Q. Integrating multi-cohort machine learning and clinical sample validation to explore peripheral blood mRNA diagnostic biomarkers for prostate cancer. *Cancer Cell Int.* **2025**, 25, 155.
[125] Zaheer, A. N.; Farhan, M.; Min, G.; Alotaibi, F. A.; Alnftai, M. M. Attention-enhanced hybrid U-Net for prostate cancer grading and explainability. *Sci. Rep.* **2025**, 15, 34038.
[126] Zhou, H.; Xie, M.; Shi, H.; Shou, C.; Tang, M.; Zhang, Y.; Hu, Y.; Liu, X. Integrating multimodal imaging and peritumoral features for enhanced prostate cancer diagnosis: A machine learning approach. *PLoS One* **2025**, 20, e0323752.
[127] Zhang, Z.; Yang, Q.; Shiradkar, R.; Mirtti, T.; Azamat, S.; Xuan, K.; Xu, J.; Madabhushi, A. A deep learning derived prostate zonal volume-based biomarker from T2-weighted MRI to distinguish between prostate cancer and benign prostatic hyperplasia. *Med. Phys.* **2025**, 52, e18053.
[128] Zhang, C.; Wang, Z.; Shang, P.; Zhou, Y.; Zhu, J.; Xu, L.; Chen, Z.; Yu, M.; Zang, Y. Combining multi-parametric MRI radiomics features with tumor abnormal protein to construct a machine learning-based predictive model for prostate cancer. *Sci. Rep.* **2025**, 15, 22816.
[129] Zarei, A.; Mazrooei Rad, E.; Salmani Bajestani, S.; Zendehbad, S. A. Providing a Prostate Cancer Detection and Prevention Method With Developed Deep Learning Approach. *Prostate Cancer* **2025**, 2025, 2019841.
[130] Telecan, T.; Chioran, A.; Sipos-Lascu, R.; Caraiani, C.; Boca, B.; Hendea, R. M.; Buliga, T.; Andras, I.; Crisan, N.; Lupsor-Platon, M. ISUP Grade Prediction of Prostate Nodules on T2WI Acquisitions Using Clinical Features, Textural Parameters and Machine Learning-Based Algorithms. *Cancers* **2025**, 17, 2035.
[131] Stojadinovic, M.; Stojadinovic, M.; Jankovic, S. Predicting intermediate-risk prostate cancer using machine learning. *Int. Urol. Nephrol.* **2025**, 57, 123–130.
[132] Srivastava, V.; Prabhu, A.; Nedungatt, S.; Damodara, K. V.; Lal, S.; Kini, J. An Efficient Parallel Branch Network for Multi-Class Classification of Prostate Cancer From Histopathological Images. *Int. J. Imaging Syst. Technol.* **2025**, 35, e70000.
[133] Yuan, Y.; Ahn, E.; Feng, D.; Khadra, M.; Kim, J. Z-SSMNet: Zonal-aware Self-supervised Mesh Network for prostate cancer detection and diagnosis with Bi-parametric MRI. *Comput. Med. Imaging Graph.* **2025**, 122, 102510.
[134] Xu, Y.; Wang, R.; Fang, Z.; Tang, J. Feasibility study of AI-assisted multi-parameter MRI diagnosis of prostate cancer. *Sci. Rep.* **2025**, 15, 10550.
[135] Wu, Q.; Wu, C.; Zhang, M.; Yang, J.; Zhang, J.; Jin, Y.; Du, Y.; Sun, X.; Jin, L.; Wang, K.; et al. Diagnosing prostate cancer in the PSA gray zone through machine learning and transrectal ultrasound

video. *J. Men's Health* **2025**, 21(5), 46–55.
[136] Qiu, J.; Chen, Q.; Lan, W.; Cao, J. Multichannel Contribution Aware Network for Prostate Cancer Grading in Histopathology Images. *J. Med. Imaging Health Inform.* **2025**, 15, 1–12.
[137] Saitta, C.; Buff, N. M.; Avolio, P.; Beatrici, E.; Paciotti, M.; Lazzeri, M.; Fasulo, V.; Cella, L.; Garofano, G.; Piccolini, A.; et al. Machine learning driven diagnostic pathway for clinically significant prostate cancer: the role of micro-ultrasound. *World J. Urol.* **2025**, 43, 487.
[138] Sadeghi, M. H.; Bagheri, H.; Rajaeinejad, M.; Ardalan, M. A.; Karami, I.; Sadeghi, S.; Mosadeghkhah, A.; Sina, S.; KhajehRahim, F.; Sheiki, M. Enhancing Prostate Cancer Detection: Integrating Multiparametric Magnetic Resonance Imaging and 68Ga-prostate-specific Membrane Antigen Positron Emission Tomography/Computed Tomography with Machine Learning. *J. Med. Phys.* **2025**, 50, 329–336.
[139] Rotteveel, A.; Lee, W.-Y.; Kountouri, Z.; Stefanou, N.; Kivell, H.; Gluck, C.; Zhang, S.; Mershin, A. Towards robust medical machine olfaction: Debiasing GC-MS data enhances prostate cancer diagnosis from urine volatiles. *PLoS One* **2025**, 20, e0314742.
[140] Rodrigues, A. C.; de Almeida, J. G.; Rodrigues, N.; Moreno, R.; Verde, A. S. C.; Galvão, A. M.; Bilreiro, C.; Santiago, I.; Ip, J.; Belião, S.; et al. Improving Clinically Significant Prostate Cancer Detection with a Multimodal Machine Learning Approach: A Large-Scale Multicenter Study. *Radiol. Imaging Cancer* **2025**, 7, e240507.
[141] Nissler, D.; Reimers-Kipping, S.; Ingwersen, M.; Niekrenz, F.; Berger, F.; Theis, B.; Gassler, N.; Grimm, M.-O.; Teichgräber, U.; Hielscher, F.; et al. Artificial Intelligence-Assisted Biparametric MRI for Detecting Prostate Cancer—A Comparative Multireader Multicase Accuracy Study. *Diagnostics* **2025**, 15, 657.
[142] Mulligi, N.; Billie, A.; Ji, X.; Szolnoky, K.; Olsson, H.; Titus, M.; Gonzalez, G. M.; Boman, S. E.; Valkonen, M.; Gudlaugsson, E.; et al. Development and retrospective validation of an artificial intelligence system for diagnostic assessment of prostate biopsies: study protocol. *BMJ Open* **2025**, 15, e097591.
[143] Prabhu, A.; Nedungatt, S.; Lal, S.; Kini, J. ProsGradNet: An effective and structured CNN approach for prostate cancer grading from histopathology images. *Biomed. Signal Process. Control* **2025**, 107626.
[144] Pausch, A. M.; Fillebock, V.; Elsner, C.; Rupp, N. J.; Eberli, D.; Hötker, A. M. Ultra-fast biparametric MRI in prostate cancer assessment: Diagnostic performance and image quality compared to conventional multiparametric MRI. *Eur. J. Radiol. Open* **2025**, 14, 100635.
[145] Oka, R.; Li, B.; Kato, S.; Utsumi, T.; Endo, T.; Kamiya, N.; Nakaguchi, T.; Suzuki, H. Computer-aided diagnosis based on 3D deep convolutional neural network system using novel 3D magnetic resonance imaging sequences for high-grade prostate cancer. *Curr. Urol.* **2025**, 19, 309–313.
[146] Lou, W.; Chen, P.; Wu, C.; Liu, Q.; Zhou, L.; Zhang, M.; Tu, J.; Hu, Z.; Lv, C.; Yang, J.; et al. Prostate cancer classification using 3D deep learning and ultrasound video clips: a multicenter study. *Front. Oncol.* **2025**, 15, 1582035.
[147] Mitura, P.; Paja, W.; Plaza, P.; Starownik, R.; Bar, K.; Klebowski, B.; Depciuch, J. Serum or urine: Which body fluids show higher sensitivity in detection prostate cancer by Raman spectroscopy. *Spectrochim. Acta A Mol. Biomol. Spectrosc.* **2025**, 327, 125123.
[148] Miao, C.; Yao, F.; Fang, J.; Tong, Y.; Lin, H.; Lu, C.; Peng, L.; Zhong, J.; Lin, Y. Exploring the role of multimodal [18F]F-PSMA-1007 PET/CT and multiparametric MRI data in predicting ISUP grading of primary prostate cancer. *Eur. J. Nucl. Med. Mol. Imaging* **2025**, 52, 2087–2095.
[149] Luo, Z.; Li, J.; Wang, K.; Li, S.; Qian, Y.; Xie, W.; Wu, P.; Wang, X.; Han, J.; Zhu, W.; et al. Study of AI algorithms on mpMRI and PHI for the diagnosis of clinically significant prostate cancer. *Urol. Oncol.* **2025**, 43, 527.e17–527.e24.
[150] Lu, X.; Guo, Y.; Zhang, S.; Yuan, Y.; Wang, C.-C.; Shen, Z.; Liu, S. Deep Augmented Metric Learning Network for Prostate Cancer Classification in Ultrasound Images. *IEEE J. Biomed. Health Inform.* **2025**, 29, 1234–1245.

[151] Li, Y.; Zhou, X.; Zhang, X.; Zhang, M.; Sun, S.; Gai, X.; Li, G. Bi-parametric MRI radiomic model for prostate cancer diagnosis: value of intralesional and perilesional radiomics. *Acta Radiol.* **2025**, 66, 639–649.
[152] Liu, Y.; Wu, J.; Ni, X.; Zheng, Q.; Wang, J.; Shen, H.; Wang, L.; Yang, R.; Weng, X. Machine learning based on automated 3D radiomics features to classify prostate cancer in patients with prostate-specific antigen levels of 4–10 ng/mL. *Transl. Androl. Urol.* **2025**, 14, 101–112.
[153] Liu, X.; Liu, R.; He, H.; Yan, Y.; Zhang, L.; Zhang, Q. Multi-regional multiparametric deep learning radiomics for diagnosis of clinically significant prostate cancer. *J. Imaging Inform. Med.* **2025**, 38, 1–13.
[154] Liu, J.; Harewood, L.; Bagguley, D.; Dundee, P.; Mirmilstein, G.; Murphy, D. G.; Chan, Y.; Moon, D.; Kearns, P.; Satasivam, P.; et al. Early results from the CONFIRM trial: utility of prostate-specific membrane antigen positron emission tomography/computed tomography in active surveillance for prostate cancer. *Eur. Urol. Oncol.* **2025**, 8, 1118–1125.
[155] Ling, C.; Tao, N.; Maimaitiyimin, A.; Zhang, Y.; Yao, M.; Pu, H.; Li, X.; Wang, Y.; An, H. Diagnostic systematic review and meta-analysis of machine learning in predicting biochemical recurrence of prostate cancer. *Sci. Rep.* **2025**, 15, 28378.
[156] Kim, E. H.; Jing, H.; Utt, K. L.; Vetter, J. M.; Weimholt, R. C.; Bullock, A. D.; Klim, A. P.; Bergeron, K. A.; Frankel, J. K.; Smith, Z. L.; et al. An artificial intelligence model using diffusion basis spectrum imaging metrics accurately predicts clinically significant prostate cancer. *J. Urol.* **2025**, 213, 778–786.
[157] Li, S.-F.; Zhao, J.-G.; Jiang, C.-Y.; Wang, S.-Y.; Liu, S.-Y.; Zhang, Y.-J.; Zeng, H.; Zhao, F.-J. Development and validation of an interpretable machine learning model for predicting Gleason score upgrade in prostate cancer. *Transl. Androl. Urol.* **2025**, 14, 1631–1644.
[158] Lee, Y. J.; Moon, H. W.; Choi, M. H.; Jung, S. E.; Park, Y. H.; Lee, J. Y.; Kim, D. H.; Rha, S. E.; Kim, S. H.; Lee, K. W.; et al. MRI-based deep learning algorithm for assisting clinically significant prostate cancer detection: a bicenter prospective study. *Radiology* **2025**, 314, e232788.
[159] Lai, C.; Hu, Z.; Li, Z.; Wu, Z.; Li, K.; Li, L.; Liu, H.; Shi, J.; Zhou, Y.; Xu, K.; et al. A machine learning-based screening model for the early detection of prostate cancer developed using serum microRNA data from a mixed cohort of 8,741 participants. *Discov. Oncol.* **2025**, 16, 1333.
[160] Gupta, A.; Lal, H.; Kumar, D.; et al. Integration of magnetic resonance imaging and deep learning for prostate cancer detection: a systematic review. *Am. J. Clin. Exp. Urol.* **2025**, 13, 69–91.
[161] Giganti, F.; Moreira da Silva, N.; Yeung, M.; Davies, L.; Frary, A.; Ferrer Rodriguez, M.; Sushentsev, N.; Ashley, N.; Andreou, A.; Bradley, A.; et al. AI-powered prostate cancer detection: a multi-centre, multi-scanner validation study. *Eur. Radiol.* **2025**, 35, 1234–1245.
[162] Johnson, P. M.; Dutt, T.; Ginocchio, L. A.; Saimbhi, A. S.; Umapathy, L.; Block, K. T.; Sodickson, D. K.; Chopra, S.; Tong, A.; Chandarana, H. Prostate cancer risk stratification and scan tailoring using deep learning on abbreviated prostate MRI. *J. Magn. Reson. Imaging* **2025**, 61, 856–864.
[163] Jiang, J.; Fan, Z.; Jiang, S.; Chen, X.; Guo, H.; Dong, S.; Jiang, T. Interpretable multimodal deep learning model for predicting post-surgical International Society of Urological Pathology grade in primary prostate cancer. *Eur. J. Nucl. Med. Mol. Imaging* **2025**, 52, 3730–3743.
[164] Harmanani, M.; Wilson, P. F. R.; To, M. N. N.; Gilany, M.; Jamzad, A.; Fooladgar, F.; Wodlinger, B.; Abolmaesumi, P.; Mousavi, P. TRUSWorthy: toward clinically applicable deep learning for confident detection of prostate cancer in micro-ultrasound. *Int. J. Comput. Assist. Radiol. Surg.* **2025**, 20, 1193–1200.
[165] Hamid, A. R. A. H.; Harahap, A. S.; Miranda, M. E.; Gibran, K.; Shabrina, N. H. Artificial intelligence for enhanced diagnostic precision of prostate cancer. *Med. J. Indones.* **2025**, 34, 189–200.
[166] Deniffel, D.; Perlis, N.; Ghai, S.; Salinas-Miranda, E.; Namdar, K.; Klotz, L. H.; Zlotta, A.; Finelli, A.; Haider, M. A. Optimizing biopsy decisions in PI-RADS 3 lesions: cross-institutional validation of a local clinical risk model. *Eur. Radiol.* **2025**, 35, 1234–1245.
[167] Geng, Y.; Zhang, X.; Zhang, M.; Li, J.; Yang, M.; Tian, J.; Ma, X. Significance of multi-task deep learning neural networks for diagnosing clinically significant prostate cancer in plain abdominal CT. *Front. Oncol.* **2025**, 15, 1543230.

[168] Du, Y.; Zhu, A.; Zhang, M.; Qiu, X.; Wang, Z.; Hu, L. Evaluation of a PSA and transrectal prostate ultrasound video-based machine learning model as a tool for prostate cancer diagnosis. *Front. Oncol.* **2025**, 15, 1590396.
[169] Du, X.; Hao, S.; Olsson, H.; Kartasalo, K.; Mulligi, N.; Rai, B.; Menges, D.; Heintz, E.; Egevad, L.; Eklund, M.; et al. Effectiveness and cost-effectiveness of artificial intelligence-assisted pathology for prostate cancer diagnosis in Sweden: a microsimulation study. *Eur. Urol. Oncol.* **2025**, 8, 80–86.
[170] Dimitriadis, A.; Osuala, R.; Kessler, D.; Diaz, O.; Mazzetti, S.; Regge, D.; Papanikolaou, N.; Fotiadis, D.; Tsiknaki, M.; Marias, K.; et al. Assessing cancer presence in prostate MRI using multi-encoder cross-attention networks. *Diagnostics* **2025**, 15, 789.
[171] Zaridis, D. I.; Pezoulas, V. C.; Mylona, E.; Kalantzopoulos, C. N.; Tachos, N. S.; Tsiknakis, N.; Matsopoulos, G. K.; Regge, D.; Papanikolaou, N.; Tsiknakis, M.; et al. Simplatab: An Automated Machine Learning Framework for Radiomics-Based Bi-Parametric MRI Detection of Clinically Significant Prostate Cancer. *Bioengineering* **2025**, 12, 242.
[172] Antonutti, D.; De Nardin, A.; Zottin, S.; Piciarelli, C.; Foresti, G. L. Multi-Modal Analysis of Bi-Parametric MRI Slices for Lesion Detection in Prostate Cancer Screening. *IEEE Access* **2025**, 13, 112234–112246.
[173] Debs, N.; Routier, A.; Bône, A.; Rohe, M.-M. Evaluation of a deep learning prostate cancer detection system on biparametric MRI against radiological reading. *Eur. Radiol.* **2025**, 35, 3134–3143.
[174] Chen, Y.; Li, F.; Dai, Z.; Liu, Y.; Huang, S.; Cheng, Q. Supervised contrastive loss helps uncover more robust features for photoacoustic prostate cancer identification. *Front. Oncol.* **2025**, 15, 1592815.
[175] Arafa, M. A.; Farhat, K. H.; Aly, S. F.; Khan, F. K.; Mokhtar, A.; Althunayan, A. M.; Al-Taweel, W.; Al-Khateeb, S. S.; Azhari, S.; Rabah, D. M. Prediction of prostate biopsy outcomes at different cut-offs of prostate-specific antigen using machine learning: a multicenter study. *World J. Urol.* **2025**, 43, 8.
[176] Ayyildiz, H.; Ince, O.; Korkut, E.; Dagoglu Kartal, M. G.; Tunaaci, A.; Erturk, S. M. Machine learning models for discriminating clinically significant from clinically insignificant prostate cancer using bi-parametric magnetic resonance imaging. *Diagn. Interv. Radiol.* **2025**, 31, 1–8.
